# Synthesis of Purine-1,4,7,10-Tetraazacyclododecane Conjugate and Its Complexation Modes with Copper(II)

**DOI:** 10.3390/molecules30071612

**Published:** 2025-04-04

**Authors:** Aleksejs Burcevs, Gediminas Jonusauskas, Irina Novosjolova, Māris Turks

**Affiliations:** 1 Institute of Chemistry and Chemical Technology, Faculty of Natural Sciences and Technology, Riga Technical University, P. Valdena Str. 3, LV-1048 Riga, Latvia; 2 Laboratoire Ondes et Matière d’Aquitaine, Bordeaux University, UMR CNRS 5798, 351 Cours de la Libération, 33405 Talence, France

**Keywords:** purines, fluorescence, metal ion complexation, synthesis, cyclen

## Abstract

Purine-1,4,7,10-tetraazacyclododecane (cyclen) conjugate was designed to study its Cu^2+^ ions complexation capability. Several synthetic approaches were tested to achieve the target compound. The optimal approach involved stepwise modifications of purine *N*9, *C*8, and *C*6 positions that, in nine consecutive steps, provided purine–cyclen conjugate. The synthetic sequence involved Mitsunobu-type alkylation at *N*9 and iodination at *C*8, followed by Stille, S_N_Ar, CuAAC, and alkylation reactions. The designed purine–cyclen conjugate is able to complex Cu^2+^ ions in both the cyclen part and between the purine *N*7 and triazole *N*2 positions. The complexation pattern and equilibrium were studied using the NMR titration technique in MeCN-d_3_ and absorption spectra.

## 1. Introduction

Purines have a broad range of applications in medicine and materials science due to their wide biological [1,2,3,4] and photophysical [5,6,7,8,9,10,11,12] properties. Therefore, the importance of the development of new synthetic approaches towards intended substitution into purine is high.

**Figure 1:**
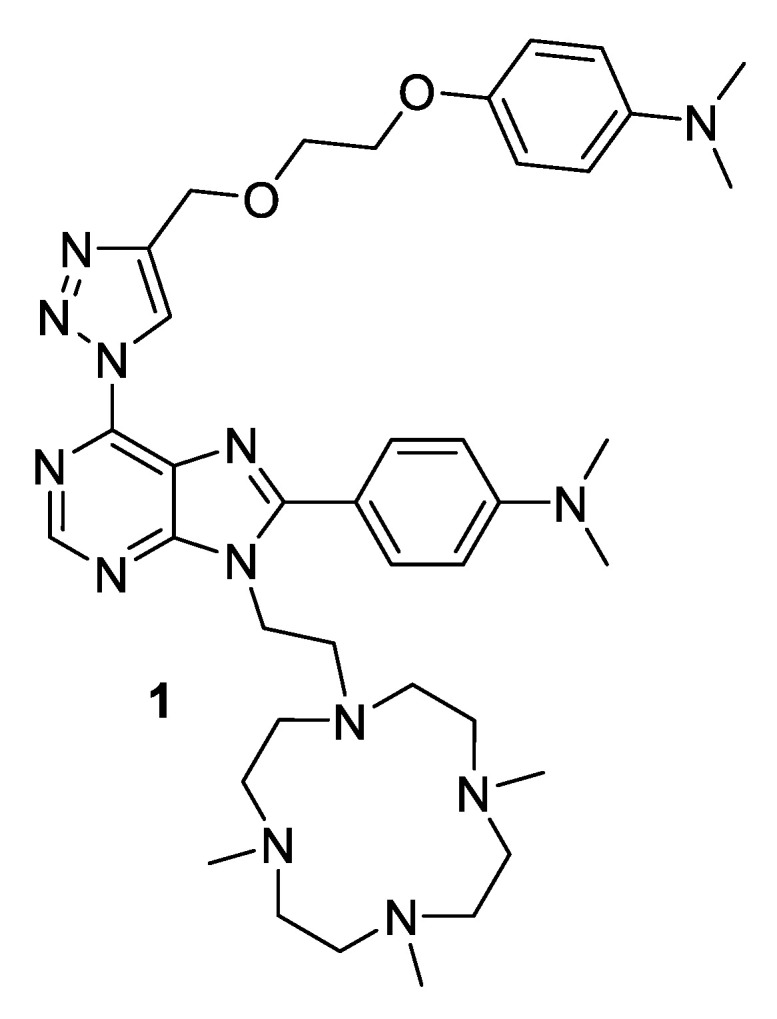
The structure of purine–cyclen conjugate **1** as a potential photocatalyst.

In this study, we designed purine–cyclen conjugate **1** as a potential photocatalyst (Figure 1). The incorporation of redox-active transition metals into organic light-absorbing molecular structures makes it possible to use light to change the oxidation state of metal ions. Cyclen derivative is an attractive moiety for derivatization due to its great ability to complex such metal ions as Cu^2+^, Ni^2+^, Pd^2+^, and Zn^2+^ [13,14,15,16,17,18]. The photoinitiated electron transfer may be easily controlled and directed toward a site containing metal ions. Moreover, the use of available molecular building blocks using well-developed synthetic methods makes the molecular construction easily adapted for specific metal ions and targeted chemical reactions. The ON/OFF switching possibility of the catalytically active state largely increases the flexibility in the control of the reaction.

Our previous work [19] inspired us to create a new structural design of the photocatalyst based on the purine core. Instead of using benzophenone with its complicated excited state behavior, including unwanted side reactions, the ground state association between dimethylaniline electron donor and strongly absorbing antenna, bearing purine, will ensure efficient light absorption and electron transfer between them without wasting the absorbed energy. The oxidation potentials of all molecular fragments are known to be optimal for an electron transfer until copper(II) is bound by cyclen. The formed copper(I) may be used for a wealth of chemical reactions, including the generation of H_2_O_2_, which possesses high reactivity towards biological structures and may also be employed for wastewater treatment using only sunlight.

**Scheme 1:**
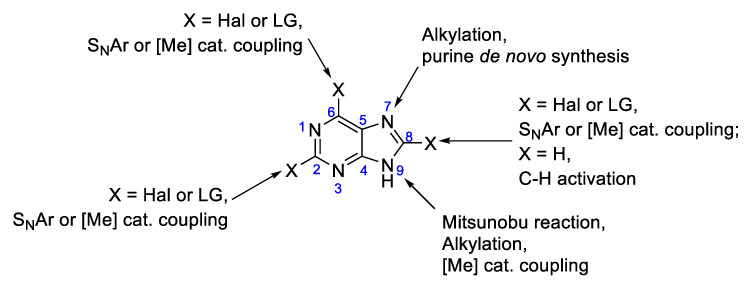
Most used approaches for derivatization of purine at *C*2, *C*6, *C*8, *N*7, and *N*9 positions.

Over the years, a wide range of methods for the derivatization of purines have been developed. Four positions in the purine core can be modified: *C*2, *C*6, *C*8, and *N*7 or *N*9. Derivatization of the C positions of the purine cycle is usually done in metal-catalyzed cross-coupling or S_N_Ar reactions (Scheme 1). In S_N_Ar reactions, halogens are the most common leaving groups in the purine, but there are reports about sulfonyl groups [20], azides [21], and C-N bonded 1,2,3-triazoles [22] acting as leaving groups as well. Various *O*- [23,24], *N*- [25,26,27], and *S*- [20,23,28] nucleophiles have been introduced to the purine ring via S_N_Ar reactions. On the other hand, the most widespread cross-coupling reactions within the purine core are Suzuki-Miyaura [29,30,31], Negishi [32,33], Stille [34,35], and Sonogashira [36,37,38] reactions. In cases of di- or trihalogenated purines, reactions occur chemo- and regioselectively [39,40], with the purine *C*6 position being the most reactive. By choosing different halogens at the purine core, it is possible to control reaction occurrence at less reactive positions with the introduction of the iodine there. It is worth noting that the proton at the purine *C*8 position is the most acidic, making it the primary target of deprotonation and C-H activation reactions [41,42,43].

In derivatizations of the *N*9 position of purine, a mixture of products is frequently formed due to the purine imidazole ring *N*7/*N*9 position tautomerism. The most used reactions for the introduction of the substituents to the *N*9 position are alkylation, Mitsunobu, and copper-catalyzed cross-coupling reactions [41,42,44]. Alkylation reactions with the formation of *N*7 product as a major product are challenging, requiring specific substrates and additives, like alkyl halides in the presence of Grignard reagents [45], glycosyl donors or *tert*-butyl halides with bis(trimethylsilyl)acetamide, TiCl_4_ or SnCl_4_ [46,47]. The most certain way of acquiring *N*7 purine derivatives is purine de novo synthesis from the corresponding pyrimidines [48] or imidazoles [49].

In addition, purine derivatives can complex metal ions and be used in medicine and as metal ion sensors. Cisplatin (*cis*-diamminedichloroplatinum(II)) is a known anticancer drug where the metal binds with the *N*7-positions of guanine bases and thus activates signal transduction pathways and apoptosis [50]. In 1971, 6-amino-, 6-chloro-, 6-hydroxy- and 6-mercaptopurines were studied for such metals as Mn^2+^, Ca^2+^, Mg^2+^, Zn^2+^, Ni^2+^, Co^2+^, and Cu^2+^ complexation in water, and it was proposed that the 1:1 complexes formed and metal coordinated to *N*7 of purine and to substituent at *C*6 position [51].

Purine–metal complexes are often studied by fluorescence because, due to the formation of complexes, fluorescence changes. Known purine-based derivatives are used as sensors for Hg^2+^ and Pd^2+^ ion detection in aqueous media [52,53] and for Cu^2+^ in cells [54,55]; upon complexation, they quench the fluorescence. Recently, there were published reports about purine nucleoside complexes for Cu^2+^ ions [56,57]. On the other hand, purine Schiff base conjugate forms complexes with Al^3+^ and increases the fluorescence [58]. 1,2,3-Triazolyl pyridine, isoquinoline, and ferrocene derivatives form complexes with Pd^2+^, Cu^2+^, Ni^2+^, Ru^2+^, Au^+^, and Ag^+^, but there is only one example in the literature of 2,6-bistriazolylpurine derivatives, which can serve as ratiometric fluorescence cation sensors, especially for the Zn^2+^ and Ca^2+^ ions [59].

The formation constant for tetramethylated cyclen and Cu^2+^ complex reaches logK 18.4 [60]. For triazole–pyridine–triazole conjugate with tridentate coordination of Cu^2+^ ions complex with logK 19.69 forms, and the complex is protonated (logβ[CuHL_2_]^3+^ [61]. On the other hand, 6-aminopurine forms a complex with Cu^2+^ ions with coordination to the *N*7 position and amino group at the *C*6 position and a formation constant logK 8.94 [62]. 3-Methyl-5-pyridin-2-yl-1,2,4-triazole forms complexes with Cu^2+^ ions in metal-to-ligand ratios 1:1 and 1:3, and their complex formation logK are 10.35 and 17.82, respectively [63]. Based on this information, the competitive complexation between cyclen moiety Cu^2+^ ions and between the *N*7 position of purine and the *N*2 position of triazole and Cu^2+^ ions may take place. In addition, the most structurally related compounds to the target compound **1** reported in the literature are purine–copper–cyclen complexes, where purine and cyclen moieties are separate ligands and are not linked within a single molecule. In these complexes, copper ions coordinate with the nitrogen atoms of the cyclen and purine *N*7, *N*9, or *N*3 positions. However, binding constants for these interactions have not been reported. Notably, these studies are focused on the chelating properties of the complexes rather than their potential photocatalytic applications [64,65].

Hence, we report here the design and synthesis of the purine derivative, which contains all the foreseen necessary substituents for metal complexation along with light absorption and electron transfer from the organic ligand to the metal center.

## 2. Results and Discussion

**Scheme 2:**
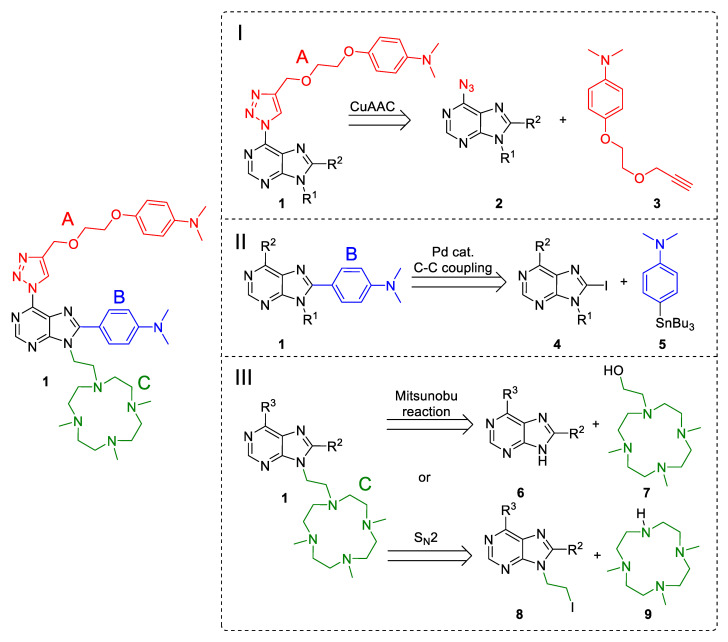
Retrosynthetic analysis toward purine–cyclen conjugate **1**.

The proposed structure consists of a purine core and three main moieties A, B, and C, which may be added in various order. The retrosynthetic analysis of the proposed structure **1** offers possible approaches toward the introduction of target building blocks (Scheme 2). Moiety A makes it possible to construct via copper-catalyzed azide–alkyne cycloaddition reaction (CuAAC) between the azido group containing purine derivative **2** and alkyne **3**. Thus, the azidation of 6-chloropurines is well-known and results in high to quantitative yields [66,67], while corresponding alkyne **3** requires a separate synthetic approach. Derivatization of the purine *C*8 position is possible to achieve via a palladium-catalyzed cross-coupling reaction. We decided to use the Stille reaction conditions due to the easy, one-step synthesis of the stannane **5** from commercially available 4-bromo-*N*,*N*-dimethylaniline [68]. The introduction of moiety C is proposed to be achieved in two ways—either via the Mitsunobu reaction between 9*H*-purine **6** and cyclen derivative **7** or using the S_N_2 reaction between substituted 9-(2-iodoethyl)-9*H*-purine **8** and cyclen derivative **9**.

**Scheme 3:**
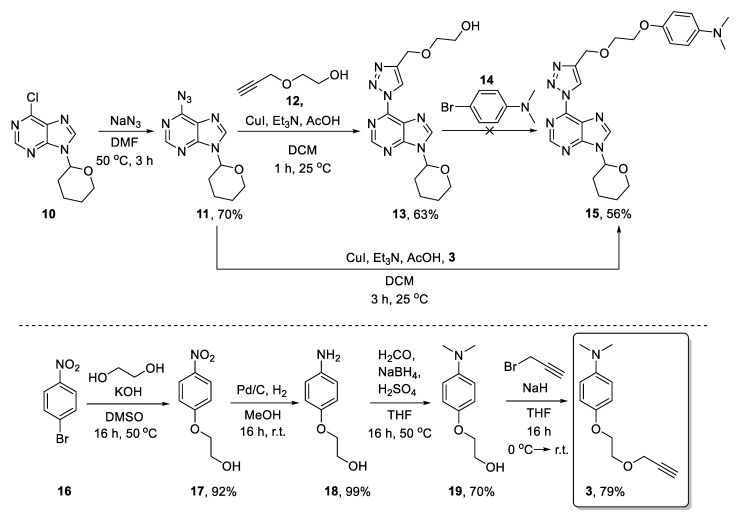
Attempts to introduce moiety A to the 6-chloro-9-tetrahydropyranyl purine **10**.

In general, we have explored several synthetic routes toward target compound **1**. The first synthetic route started with S_N_Ar reaction between 6-chloro-9-tetrahydropyranyl purine **10** [69] and sodium azide, resulting in 6-azidopurine derivative **11** in a 70% yield (Scheme 3). Next, we envisioned two approaches for the construction of moiety A at the C6 position of purine. The first approach included the CuAAC reaction of compound **11** with 2-(prop-2-yn-1-yloxy)ethan-1-ol (**12**) [70], followed by the derivatization of the free hydroxyl group of an obtained product **13**. While the CuAAC reaction successfully provided triazole derivative **13** in a 63% yield, the further derivatizations with 4-bromo-*N*,*N*-dimethylaniline (**14**) via copper-catalyzed Ullmann-type reactions were unsuccessful [71,72,73]. For this transformation, we applied previously reported reagent systems of KOH/CuI/1,10-phenanthroline/*n*-Bu_4_NI at 100 °C [71], CuI/3,4,7,8-tetramethyl-1,10-phenanthroline/Cs_2_CO_3_ at 110 °C [72] and CuI/K_3_PO_4_/8-hydroxyquinoline at 110 °C [73], but did not achieve any conversion. For the second approach, building block **3** was obtained in 4 steps from 1-bromo-4-nitrobenzene (**16**) and used in CuAAC reaction with compound **11**, resulting in a 56% yield of desired product **15**. While deprotection of the tetrahydropyranyl group at the purine *N*9 position of compound **15** was easily achieved in AcOH/H_2_O/THF mixture at 50 °C temperature overnight, further attempts to derivatize *N*9 position of this compound using the Mitsunobu (DIAD/PPh_3_/2-bromoethanol or 2-chloroethanol at 0 → 60 °C) or alkylation (NaH/1,2-dibromoethane at 0 → 100 °C) reaction procedures were not fruitful. The explanation involves the electron-poor nature of the purine system and the reduced reactivity of the *N*9 position after the introduction of the triazolyl moiety A. This encouraged us to design the second approach toward the desired compound **1**.

**Scheme 4:**
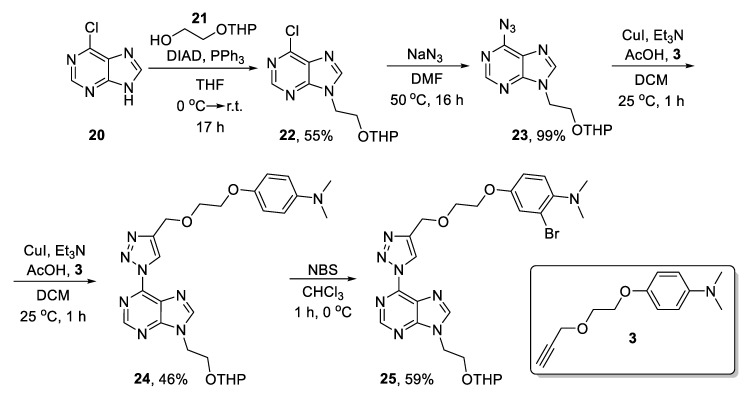
Introduction of THP-protected oxyethyl linker to 6-chloropurine (**20**), followed by a moiety A and attempts to modify the *C*8 purine position.

Next, we decided to introduce the THP-protected oxyethyl linker at the *N*9 position prior to derivatization of the *C*6 position with moiety A, keeping in mind that the cyclen part should be introduced later due to the excellent complexation ability of cyclen with transition metals [16], which would make CuAAC and cross-coupling reactions impossible. Thereby, the second synthetic route started with a Mitsunobu reaction between 6-chloropurine (**20**) and 2-(cyclohexyloxy)ethan-1-ol (**21**) [74], yielding product **22** in a 60% yield (Scheme 4). Next, the sequence of S_N_Ar and CuAAC reactions was used toward compound **24**. The following bromination attempt of the *C*8 purine position with NBS resulted in phenyl ring bromination instead, providing product **25** with a 59% yield. The use of LDA and other deprotonation reagents (NaH and *n*-BuLi) led to the deprotonation of triazole instead of the purine *C*8 position, so we concluded that the introduction of halogen to the *C*8 position and moiety B should be done prior to the derivatization of the *C*6 position.

**Scheme 5:**
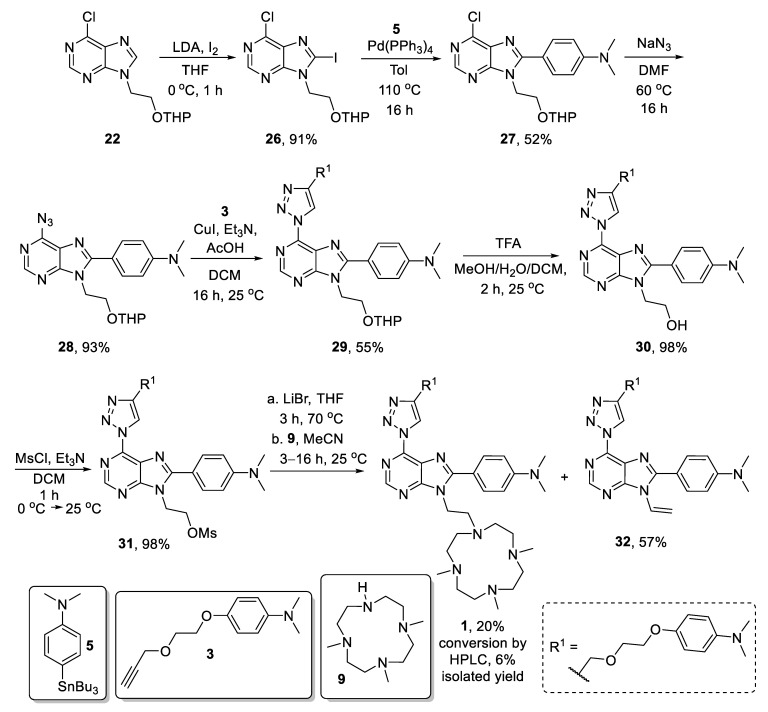
The final synthetic route toward target purine–cyclen conjugate **1**.

The final synthetic route started with the iodination of previously obtained compound **22** using the LDA and I_2_ reagent system (Scheme 5). After that, a Stille cross-coupling reaction between *N*,*N*-dimethyl-4-(tributylstannyl)aniline (**5**) and purine derivative **26** resulted in *C*8 derivatized product **27** in a 52% yield. Next, the S_N_Ar and CuAAC reaction sequence was performed to achieve compound **29**. Further, THP group cleavage with the following mesylation step resulted in almost quantitative yields in both transformations. In the first attempts, cyclen derivative **9**, which was previously obtained in three steps using literature methods [75], did not react with acquired mesylate **31** in the S_N_2 reaction. Thus, we decided to substitute the mesylate group with bromide and then perform an alkylation reaction with cyclen derivative **9**. The best conversion toward target product **1** in this reaction was 20%, with a 6% isolated yield due to the difficulties with purification stemming from the similar polarity of by-products. In addition, this transformation preferred the E2 mechanism, forming elimination product **32** as a main by-product with 57% isolated yield.

**Figure 2:**
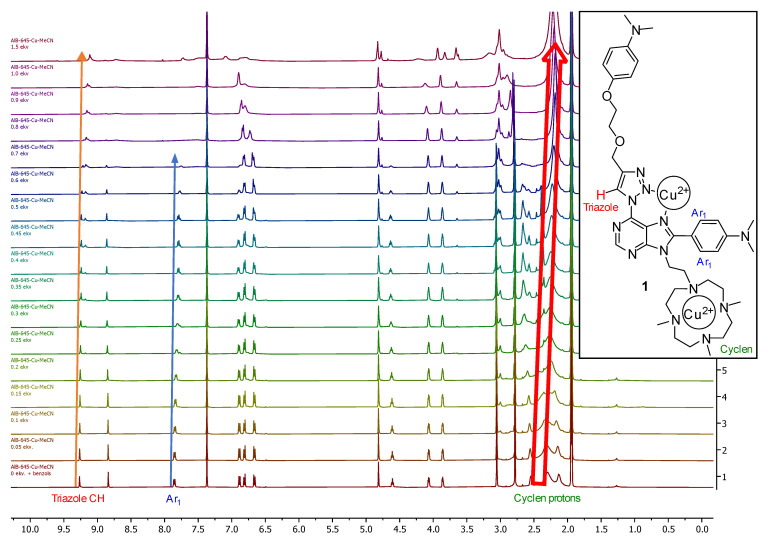
^1^H NMR titration spectra of compound **1** with Cu^2+^ ions in MeCN-*d*_3_.

With target product **1** in hand, we did preliminary studies using ^1^H NMR titration experiments (in the Supplementary Materials) to determine the complexation ability of purine–cyclen conjugate toward metal ions. Purine-cyclen conjugate **1** was titrated in MeCN-*d*_3_ with Cu^2+^ ions using Cu(ClO_4_)_2_·6H_2_O as a Cu^2+^ source and benzene as an internal standard. The corresponding spectra were taken after each 0.1 eq. addition of Cu^2+^ ions (Figure 2).

**Figure 3:**
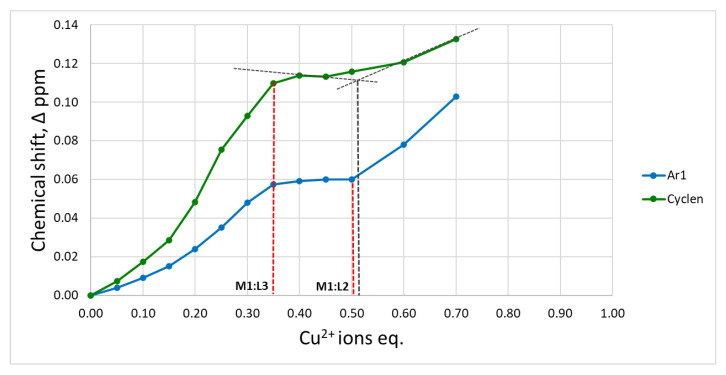
The change of the chemical shifts of Ar_1_ and cyclen proton signals in ^1^H NMR titration experiments for compound **1** with Cu^2+^ ions in MeCN-*d*_3_.

**Figure 4:**
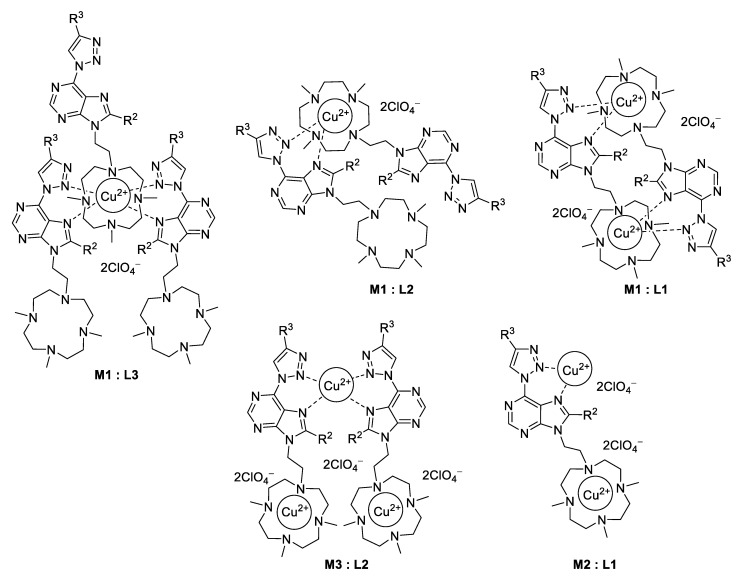
Possible Cu^2+^ complexes with purine–cyclen conjugate.

The shifts of the signals correspond to protons from triazole at the *C*6 position of purine, phenyl ring at the *C*8 position of purine, and cyclen at the *N*9 position of purine. The ^1^H NMR spectra show relatively complex behavior of the Cu^2+^ binding process by compound **1**. The most important feature representing an expected Cu^2+^ binding in cyclen is shown by a thick arrow at the values 2.0–2.5 ppm. Starting from approximately 0.7 equivalents of metal ion, all ^1^H NMR signals of cyclen assemble into a single peak at approximately 2.2 ppm, which does not shift at higher concentrations of Cu^2+^. This fact indicates that metal ion is coordinated inside the cyclen ring in a unique configuration, and all four cyclen nitrogens are equally involved in metal ion binding. An additional metal binding site between triazole and purine nitrogens is also occupied by Cu^2+,^ as is seen by triazole proton shift at approximately 9.0–9.2 ppm. The proton shifts of the other aromatic fragments of compound **1** indicate the rearrangements of electron density over the molecule during the complexation process. Based on NMR titration experiments, the possible equivalence points were determined at 0.35 and 0.50 equivalents, which corresponded to M1:L3 and M1:L2 complexes, respectively (Figure 3 and Figure 4). The competitive complexation happened between the cyclen part and the triazole *N*2 and purine *N*7 positions.

Due to the paramagnetic nature of the copper, the performance and analysis of NMR experiments and obtaining spectra at higher metal ion concentrations turn out to be difficult. Thus, the complex formation of compound **1** with Cu^2+^ and the UV–spectrophotometric titration was performed in acetonitrile (ACN) at room temperature, and it confirmed the result obtained by ^1^H NMR titration. The initial concentration of **1** was 5 × 10^−5^ mol/L. The ratio of compound **1** to Cu^2+^ was varied by adding aliquots of a solution of copper(II) perchlorate with 0.125 × 10^−6^ mol/L concentration.

**Figure 5:**
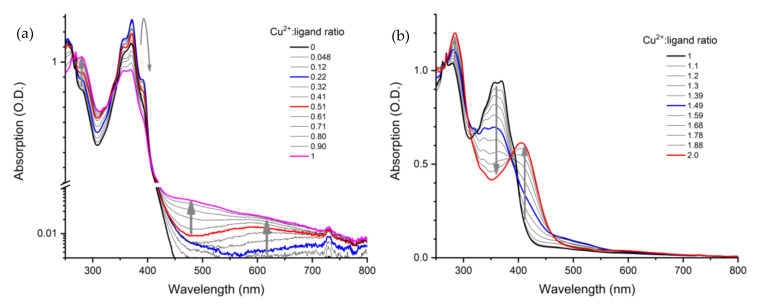
(**a**) The variation of the absorption spectra of complexes going from pure compound **1** until a 1:1 metal–ligand ratio; (**b**) the absorption spectra of complexes with compositions going from 1:1 until a 2:1 metal–ligand ratio.

**Figure 6:**
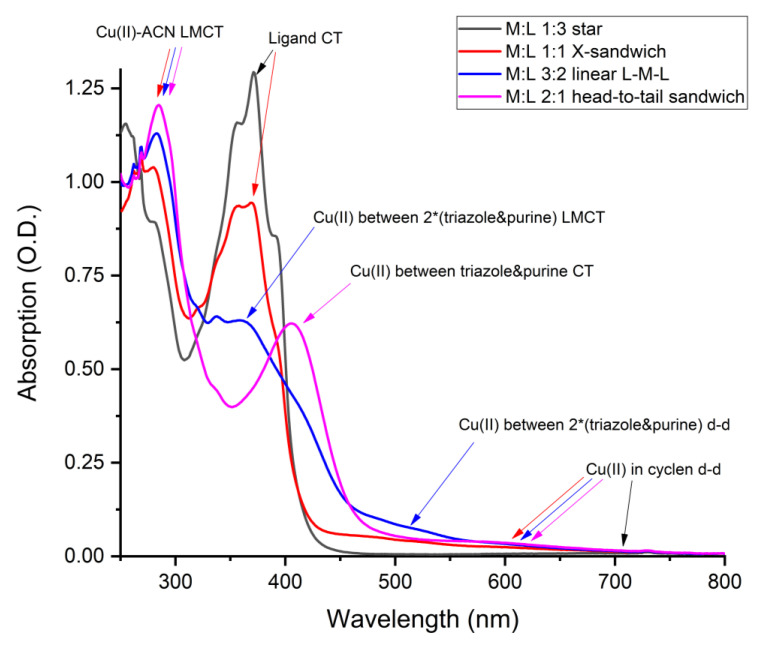
Characteristic absorption spectra extracted from spectrophotometric titration experiment indicating types of vertical transitions, compound **1**: Cu^2+^ complex compositions, and supposed geometries.

The variation of absorption spectra upon adding Cu^2+^ into the solution of **1** is quite complex, yet the step-by-step analysis of absorption spectra crossed with the data of NMR titration provides a realistic picture of the composition and geometry of the formed complexes (Figure 4, Figure 5 and Figure 6). The increase of the intensity of spectral feature at approximately 290 nm indicates the increase of the concentration of Cu^2+^ ions in the ACN solution. According to the literature [76], this absorption band may be attributed to the ligand-to-metal charge transfer (LMCT) transition between ACN and copper ion. At the concentrations corresponding to metal–ligand ratio below 1:1, the main spectral changes occur at the 450–800 nm spectral range (Figure 5a). The absorption bands observed may be attributed to the d-d transition of Cu^2+^ ions coordinated by cyclen nitrogens [15].

It is important to note that the main absorption band (including the vibronic replicas) located at 370 nm remains at the same position; only the intensity varies within 30% (Figure 5b). Such behavior may suggest that the π-conjugated electronic system of *N*,*N*-dimethylaniline-purine fragment is not affected by a metal ion. The main coordination site for Cu^2+^ is located inside cyclen until the metal–ligand ratio reaches 1:1; the ligands in excess are most probably oriented with triazole-purine fragment pointing towards Cu^2+^ inside cyclen and forming a lossy 1:3 and 1:2 complexes. After the metal–ligand ratio exceeds 1:1, the 370 nm absorption band starts to decay, causing a simultaneous rise of a new absorption band at 410 nm, which reaches its maximum at a metal–ligand ratio equal to 2:1. The formation of a new red-shifted absorption band is a typical spectroscopic signature of coordination of metal ion by an acceptor in donor-acceptor compounds; in our situation, it corresponds to the coordination of the second Cu^2+^ by triazole-purine fragment. Additionally, selected absorption spectra, together with complex compositions and supposed geometries, as well as types of vertical transitions involved [76], are presented in Figure 6.

## 3. Materials and Methods

### 3.1. General Information

Compounds **5**, **9**, **10**, **12**, and **21** were prepared according to the procedures outlined in the literature [68,69,70,74,75]. Compounds **17**–**19** were prepared using synthetic procedures described in this work, and their ^1^H spectra match the literature [77,78,79].

Commercial grade reagents and solvents were purchased from Sigma-Aldrich (Burlington, MA, USA), Fluorochem (Hadfield, UK) or BLD Pharm (Reinbek, Germany) at the highest commercial quality and used without further purification. 

The absorption spectra were recorded using the UV-VIS-NIR absorption spectrometer Varian Cary 5000 (Agilent Technologies, Santa Clara, CA, USA). Overall, more than 200 titration steps were provided to obtain the full picture of the complexation process.

### 3.2. Characterization Data for Products

#### 3.2.1. 2-(4-Nitrophenoxy)ethan-1-ol (**17**)

KOH (26.7 g, 0.48 mol, 4.0 eq.) was added in portions to abs. ethylene glycol (200 mL) at 0 °C, and the solution was stirred for 30 min at 0 °C. Then, the reaction mixture was warmed up to 25 °C and stirred until KOH was fully dissolved. Meanwhile, 1-bromo-4-nitrobenzene (**16**) (24.0 g, 0.12 mol, 1.0 eq.) was dissolved in abs. DMSO (200 mL) was then added to the KOH solution and stirred at 50 °C for 30 min. An additional abs. DMSO (40 mL) was added until everything was dissolved, and the reaction was stirred overnight at 50 °C for 16 h. The final reaction mixture was poured into cold water (1.5 L), and the formed precipitate was filtered (13.53 g of **17**). Filtrate was extracted with DCM (3 × 100 mL). The organic phase was washed with brine (100 mL), dried over anhydrous Na_2_SO_4_, filtered, evaporated in a vacuum, and then lyophilized from the remaining DMSO. After lyophilization, the solid was dissolved in a minimum amount of DCM, added to the cold hexane (100 mL), and the resulting precipitate was filtered (6.4 g of **17**). Beige amorphous solid, yield: 19.93 g, 92%. ^1^H-NMR spectrum is consistent with the literature [77].

#### 3.2.2. 2-(4-Aminophenoxy)ethan-1-ol (**18**)

Compound **17** (13.5 g, 73.77 mmol, 1.0 eq.) and 10% palladium on carbon (1.35 g) were suspended in MeOH (300 mL) and H_2_ gas was bubbled through for 6 h at 25 °C. The reaction was controlled with HPLC. The reaction mixture was filtered through a celite pad and evaporated, providing product **18** (yield: 11.12 g, 99%) as a brown oil. HPLC: t*_R_* = 1.47 min, eluent E_1_. ^1^H-NMR spectrum is consistent with the literature [78].

#### 3.2.3. 2-(4-(Dimethylamino)phenoxy)ethan-1-ol (**19**)

Compound **18** (11.12 g, 72.7 mmol, 1.0 eq.) and NaBH_4_ (16.5 g, 436.2 mmol, 6.0 eq.) were suspended in abs. THF (250 mL) and then slowly added to the mixture of H_2_SO_4_ (3M, 28.8 mL) and H_2_CO (37%, 21.6 mL) at 0 °C. The resulting mixture was left to stir for 2 h at 25 °C, then 4 h at 50 °C. The reaction mixture was basified with 2M KOH solution till pH = 11 and extracted with DCM (2 × 100 mL). The organic phase was washed with brine (2 × 100 mL), dried over anhydrous Na_2_SO_4_, filtered and evaporated. Silica gel column chromatography (EtOH/Tol, gradient 0% → 7%) provided product **19** (yield: 9.16 g, 70%) as a brown amorphous solid. R_f_ = 0.73 (Tol/EtOH = 5:1). ^1^H-NMR spectrum is consistent with the literature [79].

#### 3.2.4. *N*,*N*-Dimethyl-4-(2-(prop-2-yn-1-yloxy)ethoxy)aniline (**3**)

To the solution of compound **19** (9.16 g, 50.5 mmol, 1.0 eq.) in abs. THF (100 mL) at 0 °C, 60% NaH (3.03 g, 75.8 mmol, 1.5 eq.) was added in small portions over 30 min, then warmed until 25 °C. 80 wt% propargyl bromide in toluene (8.18 mL, ρ = 1.38 g/cm^3^, 75.9 mmol, 1.5 eq.) was added, and the reaction was left to stir for 16 h at 25 °C. The reaction mixture was quenched with water (50 mL) and extracted with DCM (2 × 100 mL); the organic phase was washed with brine (100 mL), dried over anhydrous Na_2_SO_4_, filtered, and evaporated. Silica gel column chromatography (EtOH/Tol, gradient 0% → 20%) provided product **3** (yield: 8.73 g, 79%) as a brown amorphous solid. R_f_ = 0.67 (EtOH/Tol = 5:1). HPLC: t*_R_* = 1.52 min, eluent E_1_. IR (KBr) ν (cm^−1^): 2923, 2852, 1513, 1455, 1352, 1242, 1104, 1035, 947, 817, 704. ^1^H-NMR (500 MHz, CDCl_3_) δ (ppm): 6.87 (d, 2H, ^3^*J* = 9.0 Hz, 2 × H-Ar), 6.73 (d, 2H, ^3^*J* = 9.0 Hz, 2 × H-Ar), 4.27 (s, 2H, (-CH_2_-)), 4.10 (t, 2H, ^3^*J* = 4.4 Hz, (-CH_2_-)), 3.87 (t, 2H, ^3^*J* = 4.4 Hz, (-CH_2_-)), 2.87 (s, 6H, 2 × (-CH_3_)), 2.45 (s, 1H, CH(alkynyl)). ^13^C NMR (126 MHz, CDCl_3_) δ (ppm): 151.1, 146.2, 115.9, 114.8, 79.7, 74.8, 68.6, 68.1, 58.7, 41.8. HRMS (ESI) m/z: [M + H]^+^ Calculated for C_13_H_18_NO_2_ 220.1332; Found 220.1330 (0.91 ppm).

#### 3.2.5. 6-Azido-9-(Tetrahydro-2*H*-pyran-2-yl)-9*H*-purine (**11**)

To a solution of 10 [69] (7.0 g, 29.33 mmol, 1.0 eq.) in DMF (240 mL) NaN_3_ (3.81 g, 58.66 mmol, 2 eq.) was added, and the reaction mixture was stirred at 50 °C for 3 h. Then, it was evaporated and dried in a vacuum. The resulting solid was suspended in DCM (100 mL) and washed with 5% aqueous LiCl solution (2 × 100 mL), water (2 × 200 mL), and brine (100 mL). The organic phase was dried over anhydrous Na_2_SO_4_, filtered, and evaporated, providing compound 11 (yield: 5.06 g, 70%) as a beige amorphous solid. HPLC: t*_R_* = 3.00 min, eluent E_1_. IR (KBr) ν (cm^−1^): 2917, 2852, 2134, 1640, 1374, 1345, 1228, 1083, 1043, 967, 915. ^1^H-NMR (500 MHz, DMSO-d_6_) δ (ppm): 10.12 (s, 1H, H-C(2)), 8.87 (s, 1H, H-C(8)), 5.90 (d, 1H, ^3^*J* = 11.2 Hz, H_a_-C), 4.05 (d, 1H, ^3^*J* = 11.2 Hz, H_e_-C), 3.77 (td, 1H, ^2^*J* = ^3^*J* = 11.2 Hz, ^3^*J* = 4.1 Hz, H_e_-C), 2.42–2.35 (qd, 1H, ^2^*J* = ^3^*J* = 11.2 Hz, ^3^*J* = 4.1 Hz, H_b_-C), 2.09–1.98 (m, 2H, H_b_-C, H_c_-C), 1.87–1.74 (m, 1H, H_c_-C), 1.68–1.58 (m, 2H, (-CH_2_-)). ^13^C-NMR (126 MHz, DMSO-d_6_) δ (ppm): 145.4, 142.5, 141.5, 136.0, 119.9, 82.0, 67.8, 30.0, 24.4, 22.2. HRMS (ESI) m/z: [M + H]^+^ Calculated for C_10_H_12_N_7_O 246.1098; Found 246.1093 (2.03 ppm).

#### 3.2.6. 2-({1-[9-(Tetrahydro-2*H*-pyran-2-yl)-9*H*-purin-6-yl]-1*H*-1,2,3-triazol-4-yl}methoxy)ethan-1-ol (**13**)

General method A for CuAAC reactions: To a solution of **11** (1.0 g, 4.08 mmol, 1.0 eq.), CuI (0.14 g, 0.73 mmol, 0.18 eq.), AcOH (0.26 mL, ρ = 1.05 g/cm^3^, 4.49 mmol, 1.1 eq.) and Et_3_N (0.63 mL, ρ = 0.73 g/cm^3^, 8.16 mmol, 2 eq.) in DCM (40 mL) 2-(prop-2-yn-1-yloxy)ethanol (0.82 g, 0.90 mmol, 1.5 eq.) (**12**) [70] was added and the reaction mixture was stirred isolated from the daylight for 1 h at 25 °C. Then, the reaction mixture was poured into water (50 mL) and extracted with DCM (3 × 50 mL). The combined organic phase was washed with aqueous NaHS (2 × 30 mL) and brine (50 mL), dried over anhydrous Na_2_SO_4_, filtered, and evaporated. Silica gel column chromatography (DCM/EtOH, gradient 0% → 15%) provided product **13** (yield: 881 mg, 63%) as a white amorphous solid. R_f_ = (0.48 DCM/EtOH = 10:1). HPLC: t*_R_* = 2.16 min, eluent E_1_. IR (KBr) ν (cm^−1^): 3370, 3111, 2924, 2862, 1616, 1574, 1462, 1335, 1212, 1082, 1014, 908, 822. ^1^H-NMR (500 MHz, CDCl_3_) δ 9.07 (s, 1H, H-C(triazole)), 8.92 (s, 1H, H-C(2)), 8.42 (s, 1H, H-C(8)), 5.86 (dd, 1H, ^2^*J* = 10.5 Hz, ^3^*J* = 2.5 Hz, H_a_-C), 4.84 (s, 2H, (-CH_2_-)), 4.23–4.18 (m, 1H, H_e_-C), 3.84–3.75 (m, 3H, H_e_-C, (-CH_2_-)), 3.74–3.70 (m, 2H, (-CH_2_-)), 2.45 (s, 1H, (-OH)), 2.26–2.17 (m, 1H, H_b_-C), 2.14–2.01 (m, 2H, H_b_-C, H_c_-C), 1.87–1.73 (m, 2H, H_c_-C, H_d_-C), 1.73–1.64 (m, 1H, H_d_-C). ^13^C-NMR (126 MHz, CDCl_3_) δ (ppm): 153.8, 152.3, 145.8, 144.9, 144.1, 123.2, 122.9, 82.6, 72.1, 69.1, 64.5, 62.0, 32.1, 24.9, 22.8. HRMS (ESI) m/z: [M + H]^+^ Calculated for C_15_H_20_N_7_O_3_ 346.1622; Found 346.1613 (2.6 ppm).

#### 3.2.7. *N*,*N*-Dimethyl-4-[2-({1-[9-(tetrahydro-2*H*-pyran-2-yl)-9*H*-purin-6-yl]-1*H*-1,2,3-triazol-4-yl}methoxy)ethoxy]aniline (**15**)

Prepared according to general method A: Compound **11** (85 mg, 0.35 mmol, 1.0 eq.), CuI (12 mg, 0.06 mmol, 0.18 eq.), AcOH (23 µL, ρ = 1.05 g/cm^3^, 0.39 mmol, 1.1 eq.), Et_3_N (54 µL, ρ = 0.73 g/cm^3^, 0.39 mmol, 1.1 eq.) and *N*,*N*-dimethyl-4-(2-(prop-2-yn-1-yloxy)ethoxy)aniline (115 mg, 0.52 mmol, 1.5 eq.), DCM (4 mL), 25 °C, 3 h. Silica gel column chromatography (Tol/EtOH, gradient 0% → 8%) provided product **15** (yield: 90 mg, 56%) as a gray amorphous solid. R_f_ = 0.49 (Tol/EtOH = 5:1). HPLC: t*_R_* = 1.93 min, eluent E_1_. IR (KBr) ν (cm^−1^): 2939, 2869, 2801, 1614, 1523, 1456, 1231, 1209, 1083, 1032, 910, 803. ^1^H-NMR (500 MHz, CDCl_3_) δ 9.11 (s, 1H, H-C(triazole)), 8.92 (s, 1H, H-C(2)), 8.42 (s, 1H, H-C(8)), 6.85 (d, 2H, ^3^*J* = 6.5 Hz, 2 × H-C(Ar)), 6.68 (d, 2H, ^3^*J* = 6.5 Hz, 2 × H-C(Ar)), 5.85 (d, 1H, ^3^*J* = 10.5 Hz, H_a_-C), 4.90 (s, 2H, (-CH_2_-)), 4.20 (d, 1H, ^3^*J* = 10.5 Hz, H_e_-C), 4.14–4.05 (m, 2H, (-CH_2_-)), 3.95–3.88 (m, 2H, (-CH_2_-)), 3.80 (t, 1H, ^3^*J* = 10.5 Hz, H_e_-C), 2.83 (s, 6H, 2 × (-CH_3_)), 2.23–2.16 (m, 1H, H_b_-C), 2.13–2.01 (m, 2H, H_b_-C, H_c_-C), 1.87–1.72 (m, 2H, H_c_-C, H_d_-C), 1.71–1.64 (m, 1H, H_d_-C). ^13^C-NMR (126 MHz, CDCl_3_) δ (ppm): 153.8, 152.3, 151.0, 146.0, 145.9, 144.8, 144.0, 123.3, 122.8, 115.8, 114.7, 82.6, 69.4, 69.0, 68.2, 64.7, 41.8, 31.9, 24.9, 22.7. HRMS (ESI) m/z: [M + H]^+^ Calculated for C_23_H_29_N_8_O_3_ 465.2357; Found 465.2355 (0.43 ppm).

#### 3.2.8. 6-Chloro-9-{2-[(Tetrahydro-2*H*-pyran-2-yl)oxy]ethyl}-9*H*-purine (**22**)

To a suspension of 6-chloropurine (**20**) (4.8 g, 31.15 mmol, 1.1 eq.), PPh_3_ (9.65 g, 36.82 mmol, 1.3 eq.) and 2-((tetrahydro-2*H*-pyran-2-yl)oxy)ethanol (**21**) [74] (4.14 g, 28.32 mmol, 1.0 eq.) in dry THF (200 mL) at 0 °C DIAD (6.12 mL, ρ = 1.03 g/cm^3^, 31.15 mmol, 1.1 eq.) was added slowly over 1 h. Then, the reaction mixture was left to stir for 16 h at 25 °C and then filtered. The filtrate was evaporated. Reverse phase silica gel column chromatography (MeOH/H_2_O, gradient 0% → 70%) provided product **22** (yield: 4.82 g, 55%) as a pale yellow oil. R_f_ = 0.84 (DCM/EtOH = 10:1). HPLC: t*_R_* = 2.15 min, eluent E_1_. IR (KBr) ν (cm^−1^): 2941, 2867, 1593, 1560, 1334, 1197, 1123, 1034, 933, 856. ^1^H-NMR (500 MHz, CDCl_3_) δ (ppm): 8.73 (s, 1H, H-C(2)), 8.28 (s, 1H, H-C(8)), 4.56–4.45 (m, 3H, H_a_-C, (-CH_2_-)), 4.11–4.05 (m, 1H, H_f_-C), 3.77–3.71 (m, 1H, H_f_-C), 3.61–3.54 (m, 1H, H_e_-C), 3.44–3.38 (m, 1H, H_e_-C), 1.77–1.62 (m, 2H, H_b_-C, H_c_-C), 1.57–1.45 (m, 4H, H_b_-C, H_c_-C, (-CH_2_-)). ^13^C-NMR (126 MHz, CDCl_3_) δ (ppm): 152.0, 151.9, 151.0, 146.5, 131.6, 99.3, 65.2, 62.6, 44.4, 30.5, 25.3, 19.5. HRMS (ESI) m/z: [M + H]^+^ Calculated for C_12_H_16_ClN_4_O_2_ 283.0956; Found 283.0950 (2.12 ppm).

#### 3.2.9. 6-Azido-9-{2-[(Tetrahydro-2*H*-pyran-2-yl)oxy]ethyl}-9*H*-purine (**23**)

To a solution of **22** (0.72 g, 2.54 mmol, 1.0 eq.) in DMF (14 mL) NaN_3_ (0.33 g, 5.08 mmol, 2 eq.) was added and the reaction mixture was stirred at 50 °C for 16 h. Then, it was evaporated and dried in a vacuum. The resulting solid was suspended in DCM (20 mL) and washed with 5% aqueous LiCl solution (2 × 20 mL), water (2 × 20 mL), and brine (20 mL). The organic phase was dried over anhydrous Na_2_SO_4_, filtered, and evaporated, providing compound **23** (yield: 0.73 g, 99%) as a white amorphous solid. HPLC: t*_R_* = 2.94 min, eluent E_1_. IR (KBr) ν (cm^−1^): 2985, 2938, 2185, 1636, 1376, 1345, 1256, 1125, 1071, 981, 872. ^1^H-NMR (500 MHz, DMSO-d_6_) δ (ppm): 10.11 (s, 1H, H-C(2)), 8.63 (s, 1H, H-C(8)), 4.68–4.55 (m, 3H, H_a_-C, (-CH_2_-)), 4.02 (ddd, 1H, ^2^*J* = 10.9 Hz, ^3^*J* = 6.5, 4.1 Hz, H_f_-C), 3.84 (ddd, 1H, ^2^*J* = 10.9 Hz, ^3^*J* = 6.5, 4.1 Hz, H_f_-C), 3.44 (ddd, 1H, ^2^*J* = 11.2 Hz, ^3^*J* = 8.4, 3.0 Hz, H_e_-C), 3.32–3.29 (m, 1H, H_e_-C), 1.64–1.48 (m, 2H, H_b_-C, H_c_-C), 1.45–1.31 (m, 4H, H_b_-C, H_c_-C, (-CH_2_-)). ^13^C NMR (126 MHz, DMSO-d_6_) δ (ppm): 145.4, 144.7, 142.3, 135.7, 119.6, 97.6, 64.6, 61.1, 44.3, 29.9, 24.8, 18.8. HRMS (ESI) m/z: [M + H]^+^ Calculated for C_12_H_16_N_7_O_2_ 290.1360; Found 290.1350 (3.45 ppm).

#### 3.2.10. *N*,*N*-Dimethyl-4-(2-{[1-(9-{2-[(Tetrahydro-2*H*-pyran-2-yl)oxy]ethyl}-9*H*-purin-6-yl)-1*H*-1,2,3-triazol-4-yl]methoxy}ethoxy)aniline (**24**)

Prepared according to general method A: Compound **23** (100 mg, 0.35 mmol, 1.0 eq.), CuI (12 mg, 0.06 mmol, 0.16 eq.), AcOH (21 µL, ρ = 1.05 g/cm^3^, 0.38 mmol, 1.1 eq.), Et_3_N (53 µL, ρ = 0.73 g/cm^3^, 0.38 mmol, 1.1 eq.) and *N*,*N*-dimethyl-4-(2-(prop-2-yn-1-yloxy)ethoxy)aniline (151 mg, 0.69 mmol, 2.0 eq.), DCM (3 mL), 25 °C, 1 h. Silica gel column chromatography (DCM/EtOH, gradient 0% → 5%) provided product **24** (yield: 81 mg, 46%) as a pale brown amorphous solid. R_f_ = 0.53 (DCM/EtOH = 20:1). HPLC: t*_R_* = 5.03 min, eluent E_2_. IR (KBr) ν (cm^−1^): 2919, 2868, 1605, 1513, 1331, 1234, 1111, 1032, 845, 823. ^1^H-NMR (500 MHz, CDCl_3_) δ (ppm): 9.11 (s, 1H, H-C(triazole)), 8.91 (s, 1H, H-C(2)), 8.39 (s, 1H, H-C(8)), 6.86 (d, 2H, ^3^*J* = 9.2 Hz, 2 × H-C(Ar)), 6.70 (d, 2H, ^3^*J* = 9.2 Hz, 2 × H-C(Ar)), 4.90 (s, 2H, (-CH_2_-)), 4.64–4.51 (m, 3H, H_a_-C, (-CH_2_-)), 4.17–4.12 (m, 1H, H_f_-C), 4.11 (dd, 2H, ^3^*J* = 6.0, 4.0 Hz, (-CH_2_-)), 3.92 (dd, 2H, ^3^*J* = 6.0, 4.0 Hz, (-CH_2_-)), 3.78 (ddd, 1H, ^2^*J* = 10.7 Hz, ^3^*J* = 7.1, 3.4 Hz, H_f_-C), 3.62–3.56 (m, 1H, H_e_-C), 3.46–3.39 (m, 1H, H_e_-C), 2.83 (s, 6H, 2 × (-CH_3_)), 1.80–1.64 (m, 2H, H_b_-C, H_c_-C), 1.59–1.45 (m, 4H, H_b_-C, H_c_-C, (-CH_2_-)). ^13^C-NMR (126 MHz, CDCl_3_) δ (ppm): 154.6, 152.1, 151.1, 147.3, 146.0, 145.9, 144.7, 123.3, 122.6, 115.8, 114.8, 99.3, 69.4, 68.2, 65.2, 64.8, 62.6, 44.4, 41.8, 30.5, 25.2, 19.4. HRMS (ESI) m/z: [M + H]^+^ Calculated for C_25_H_33_N_8_O_4_ 509.2619; Found 509.2624 (0.98 ppm).

#### 3.2.11. 2-Bromo-*N*,*N*-Dimethyl-4-{2-[(1-(9-{2-[(Tetrahydro-2*H*-pyran-2-yl)oxy]ethyl}-9*H*-purin-6-yl)-1*H*-1,2,3-triazol-4-yl)methoxy]ethoxy}aniline (**25**)

To a solution of **24** (100 mg, 0.2 mmol, 1.0 eq.) in CHCl_3_ (3 mL) at 0 °C NBS (37 mg, 0.21 mmol, 1.05 eq.) was slowly added. The reaction mixture was left to stir for 1 h at 0 °C, then diluted with CHCl_3_ to 20 mL in total, washed with water (3 × 50 mL) and brine (20 mL), then evaporated and dried in a vacuum. Silica gel column chromatography (DCM/EtOH, gradient 0% → 10%) provided product **25** (yield: 68 mg, 59%) as a brown oil. R_f_ = 0.85 (DCM/EtOH = 10:1). HPLC: t*_R_* = 5.37 min, eluent E_2_. IR (KBr) ν (cm^−1^): 2940, 2867, 1604, 1575, 1331, 1223, 1122, 1031, 991, 850. ^1^H-NMR (500 MHz, CDCl_3_) δ (ppm): 9.14 (s, 1H, H-C(triazole)), 8.94 (s, 1H, H-C(2)), 8.42 (s, 1H, H-C(8)), 7.20 (d, 1H, ^4^*J* = 2.8 Hz, H-C(Ar)), 7.02 (d, 1H, ^3^*J* = 8.9 Hz, H-C(Ar)), 6.85 (dd, 1H, ^3^*J* = 8.9 Hz, ^4^*J* = 2.8 Hz, H-C(Ar)), 4.91 (s, 2H, (-CH_2_-)), 4.66–4.51 (m, 3H, H_a_-C, (-CH_2_-)), 4.17–4.13 (m, 1H, H_f_-C), 4.12 (dd, 2H, ^3^*J* = 5.7, 3.6 Hz, (-CH_2_-)), 3.94 (dd, 2H, ^3^*J* = 5.7, 3.6 Hz, (-CH_2_-)), 3.79 (ddd, 1H, ^2^*J* = 10.7 Hz, ^3^*J* = 7.1, 3.3 Hz, H_f_-C), 3.65–3.58 (m, 1H, H_e_-C), 3.47–3.40 (m, 1H, H_e_-C), 2.71 (s, 6H, 2 × (-CH_3_)), 1.81–1.69 (m, 2H, H_b_-C, H_c_-C), 1.59–1.44 (m, 4H, H_b_-C, H_c_-C, (-CH_2_-)). ^13^C-NMR (126 MHz, CDCl_3_) δ (ppm): 155.1, 154.6, 152.2, 147.4, 145.9, 145.7, 144.8, 123.3, 122.6, 121.1, 120.2, 115.9, 114.3, 99.4, 69.1, 68.1, 65.3, 64.8, 62.7, 44.9, 44.4, 30.5, 25.3, 19.5. HRMS (ESI) m/z: [M + H]^+^ Calculated for C_25_H_32_BrN_8_O_4_ 587.1724; Found 587.1707 (2.90 ppm).

#### 3.2.12. 6-Chloro-8-iodo-9-{2-[(Tetrahydro-2*H*-pyran-2-yl)oxy]ethyl}-9*H*-purine (**26**)

To dry, degassed THF (96 mL) at −78 °C DIPEA (3.71 mL, ρ = 0.72 g/cm^3^, 26.31 mmol, 1.55 eq.) and *n*-BuLi (10.38 mL, 2.29 M, 23.77 mmol, 1.4 eq.) were added and left to stir for 30 min at −78 °C. Compound **22** (4.8 g, 16.98 mmol, 1.0 eq.) was dissolved in dry, degassed THF (48 mL), slowly added to the solution of LDA at −78 °C, and left to stir for 1 h at that temperature. I_2_ (21.5 g, 84.89 mmol, 5.0 eq.) was dissolved in dry, degassed THF (20 mL) and slowly added to the reaction mixture until it became solid. Then, the reaction was warmed up to room temperature, and saturated aqueous Na_2_S_2_O_3_·5H_2_O solution was added until the aqueous phase became colorless. The reaction mixture was washed with EtOAc (3 × 150 mL), and the combined organic phase was washed with brine (100 mL) and dried over anhydrous Na_2_SO_4_, filtered, and evaporated. Silica gel column chromatography (DCM/EtOH, gradient 0% → 5%) provided product **26** (yield: 6.34 g, 91%) as a pale brown amorphous solid. R_f_ = 0.65 (DCM/EtOH = 20:1). HPLC: t*_R_* = 4.66 min, eluent E_1_. IR (KBr) ν (cm^−1^): 2940, 2865, 1587, 1558, 1454, 1321, 1150, 1123, 1068, 1037, 948, 866. ^1^H-NMR (500 MHz, CDCl_3_) δ (ppm): 8.65 (s, 1H, H-C(2)), 4.58–4.41 (m, 3H, H_a_-C, (-CH_2_-)), 4.10 (ddd, 1H, ^2^*J* = 11.1 Hz, ^3^*J* = 6.8, 4.5 Hz, H_f_-C), 3.81 (ddd, 1H, ^2^*J* = 11.1 Hz, ^3^*J* = 6.8, 4.5 Hz, H_f_-C), 3.48 (td, 1H, ^2^*J* = 11.4 Hz, ^3^*J* = 3.3 Hz, H_e_-C), 3.41–3.34 (m, 1H, H_e_-C), 1.73–1.57 (m, 2H, H_b_-C, H_c_-C), 1.54–1.40 (m, 4H, H_b_-C, H_c_-C, (-CH_2_-)). ^13^C-NMR (126 MHz, CDCl_3_) δ (ppm): 153.1, 151.9, 149.3, 134.0, 109.1, 98.5, 64.3, 62.0, 46.6, 30.2, 25.3, 19.0. HRMS (ESI) m/z: [M + H]^+^ Calculated for C_12_H_15_ClIN_4_O_2_ 408.9923; Found 408.9913 (2.45 ppm).

#### 3.2.13. 4-(6-Chloro-9-{2-[(Tetrahydro-2*H*-pyran-2-yl)oxy]ethyl}-9*H*-purin-8-yl)-*N*,*N*-Dimethylaniline (**27**)

In the pressure flask a solution of compound **26** (825 mg, 2.02 mmol, 1.0 eq.), *N*,*N*-dimethyl-4-(tributylstannyl)aniline [68] (911 mg, 2.22 mmol, 1.1 eq.) and Pd(PPh_3_)_4_ (116 mg, 0.1 mmol, 0.05 eq.) in dry toluene (25 mL) was stirred at 110 °C for 16 h. Then, it was evaporated and dried in a vacuum. Silica gel column chromatography (DCM/EtOH, gradient 0% → 25%) provided product **27** (yield: 420 mg, 52%) as a pale orange amorphous solid. R_f_ = 0.60 (DCM/EtOH = 20:1). HPLC: t*_R_* = 5.89 min, eluent E_1_. IR (KBr) ν (cm^−1^): 2930, 2863, 1609, 1479, 1365, 1343, 1125, 1037, 978, 948, 819. ^1^H-NMR (500 MHz, CDCl_3_) δ (ppm): 8.67 (s, 1H, H-C(2)), 7.91 (d, 2H, ^3^*J* = 8.6 Hz, 2 × H-C(Ar)), 6.78 (d, 2H, ^3^*J* = 8.6 Hz, 2 × H-C(Ar)), 4.65–4.55 (m, 2H, (-CH_2_-)), 4.53–4.48 (m, 1H, H_a_-C), 4.26–4.16 (m, 1H, H_f_-C), 4.00–3.90 (m, 1H, H_f_-C), 3.56–3.47 (m, 1H, H_e_-C), 3.43–3.35 (m, 1H, H_e_-C), 3.07 (s, 6H, 2 × (-CH_3_)), 1.75–1.59 (m, 2H, H_b_-C, H_c_-C), 1.55–1.40 (m, 4H, H_b_-C, H_c_-C, (-CH_2_-)). ^13^C-NMR (126 MHz, CDCl_3_) δ (ppm): 157.8, 154.3, 152.0, 150.8, 148.7, 131.8, 131.2, 115.6, 111.8, 98.8, 64.7, 62.0, 44.7, 40.3, 30.3, 25.3, 19.2. HRMS (ESI) m/z: [M + H]^+^ Calculated for C_20_H_25_ClN_5_O_2_ 402.1691; Found 402.1685 (1.49 ppm).

#### 3.2.14. 4-(6-Azido-9-{2-[(Tetrahydro-2*H*-pyran-2-yl)oxy]ethyl}-9*H*-purin-8-yl)-*N*,*N*-Dimethylaniline (**28**)

To a solution of **27** (2.84 g, 7.07 mmol, 1.0 eq.) in DMF (140 mL) NaN_3_ (1.40 g, 21.22 mmol, 3.0 eq.) was added and the reaction mixture was stirred at 60 °C for 16 h. Then, the reaction mixture was poured into cold water (1 L), and the precipitate was filtered and washed with water (0.5 L), providing compound **28** (yield: 2.69 g, 93%) as a pale pink amorphous solid. HPLC: t*_R_* = 4.66 min, eluent E_1_. IR (KBr) ν (cm^−1^): 2939, 2886, 2025, 1608, 1478, 1344, 1202, 1126, 1073, 977, 874, 821. ^1^H-NMR (500 MHz, DMSO-d_6_) δ (ppm): 10.08 (s, 1H, H-C(2)), 7.86 (d, 2H, ^3^*J* = 8.7 Hz, 2 × H-C(Ar)), 6.86 (d, 2H, ^3^*J* = 8.7 Hz, 2 × H-C(Ar)), 4.48–4.45 (m, 2H, (-CH_2_-)), 4.48–4.43 (m, 1H, H_a_-C), 4.06 (ddd, 1H, ^2^*J* = 11.2 Hz, ^3^*J* = 6.7, 4.7 Hz, H_f_-C), 3.86 (ddd, 1H, ^2^*J* = 11.2 Hz, ^3^*J* = 6.7, 4.7 Hz, H_f_-C), 3.35–3.30 (m, 1H, H_e_-C), 3.26–3.22 (m, 1H, H_e_-C), 3.02 (s, 6H, 2 × (-CH_3_)), 1.47–1.40 (m, 2H, H_b_-C, H_c_-C), 1.37–1.24 (m, 4H, H_b_-C, H_c_-C, (-CH_2_-)). ^13^C-NMR (126 MHz, DMSO-d_6_) δ (ppm): 154.6, 151.4, 144.9, 144.0, 134.4, 130.3, 119.6, 115.6, 111.6, 97.5, 64.2, 60.8, 44.7, 39.72, 29.7, 24.7, 18.6. HRMS (ESI) m/z: [M + H]^+^ Calculated for C_20_H_25_N_8_O_2_ 409.2095; Found 409.2097 (0.49 ppm).

#### 3.2.15. 4-[6-(4-({2-[4-(Dimethylamino)phenoxy]ethoxy)methyl}-1*H*-1,2,3-triazol-1-yl)-9-{2-[(Tetrahydro-2*H*-pyran-2-yl)oxy]ethyl}-9*H*-purin-8-yl]-*N*,*N*-Dimethylaniline (**29**)

Prepared according to general method A: Compound **28** (500 mg, 1.22 mmol, 1.0 eq.), CuI (40 mg, 0.20 mmol, 0.16 eq.), AcOH (77 µL, ρ = 1.05 g/cm^3^, 1.35 mmol, 1.1 eq.), Et_3_N (187 µL, ρ = 0.73 g/cm^3^, 1.35 mmol, 1.1 eq.) and *N*,*N*-dimethyl-4-(2-(prop-2-yn-1-yloxy)ethoxy)aniline (540 mg, 2.45 mmol, 2.0 eq.), DCM (20 mL), 25 °C, 16 h. Silica gel column chromatography (DCM/EtOH, gradient 0% → 3%) provided product **29** (yield: 420 mg, 55%) as a brown amorphous solid. R_f_ = 0.43 (DCM/EtOH = 20:1). HPLC: t*_R_* = 4.15 min, eluent E_1_. IR (KBr) ν (cm^−1^): 2923, 2855, 1606, 1512, 1457, 1338, 1243, 1118, 1032, 816, 797. ^1^H-NMR (500 MHz, CDCl_3_) δ (ppm): 9.40 (s, 1H, H-C(triazole)), 8.88 (s, 1H, H-C(2)), 7.96 (d, 2H, ^3^*J* = 8.6 Hz, 2 × H-C(Ar)), 6.85 (d, 2H, ^3^*J* = 8.6 Hz, 2 × H-C(Ar)), 6.78 (d, 2H, ^3^*J* = 8.6 Hz, 2 × H-C(Ar)), 6.67 (d, 2H, ^3^*J* = 8.6 Hz, 2 × H-C(Ar)), 4.91 (s, 2H, (-CH_2_-)), 4.72–4.62 (m, 2H, (-CH_2_-)), 4.54–4.49 (m, 1H, H_a_-C), 4.25 (ddd, 1H, ^2^*J* = 11.0 Hz, ^3^*J* = 5.6, 5.2 Hz, H_f_-C), 4.10 (t, 2H, ^3^*J* = 4.7 Hz, (-CH_2_-)), 3.98 (ddd, 1H, ^2^*J* = 11.0 Hz, ^3^*J* = 5.6, 5.2 Hz, H_f_-C), 3.92(t, 2H, ^3^*J* = 4.7 Hz, (-CH_2_-)), 3.55–3.48 (m, 1H, H_e_-C), 3.40–3.34 (m, 1H, H_e_-C), 3.07 (s, 6H, 2 × (-CH_3_)), 2.82 (s, 6H, 2 × (-CH_3_)), 1.64–1.54 (m, 2H, H_b_-C, H_c_-C), 1.51–1.36 (m, 4H, H_b_-C, H_c_-C, (-CH_2_-)). ^13^C NMR (126 MHz, CDCl_3_) δ 158.4, 157.0, 152.1, 151.1, 151.0, 146.0, 145.8, 142.9, 131.2, 124.5, 122.8, 115.8, 115.5, 114.8, 111.7, 98.8, 69.1, 68.2, 64.68, 64.67, 62.0, 44.7, 41.8, 40.2, 30.3, 25.3, 19.1. HRMS (ESI) m/z: [M + H]^+^ Calculated for C_33_H_42_N_9_O_4_ 628.3354; Found 628.3383 (4.62 ppm).

#### 3.2.16. 2-{6-[4-({2-[4-(Dimethylamino)phenoxy]ethoxy}methyl)-1*H*-1,2,3-triazol-1-yl]-8-[4-(Dimethylamino)phenyl]-9*H*-purin-9-yl}ethan-1-ol (**30**)

To the mixture of compound **29** (815 mg, 1.30 mmol, 1.0 eq.) in 52 mL of MeOH/H_2_O/DCM mixture (10:2:1) TFA (0.99 mL, ρ = 1.49 g/cm^3^, 13.0 mmol, 10.0 eq.) was added at 25 °C. Then, the reaction was left stirring for 2 h, evaporated, dissolved in DCM (10 mL), and washed with aqueous NaHCO_3_ solution (10 mL) and brine (10 mL). The organic phase was dried over anhydrous Na_2_SO_4_, filtered, and evaporated, providing product **30** (yield: 689 mg, 98%) as a dark yellow amorphous solid. R_f_ = 0.68 (DCM/EtOH = 10:1). HPLC: t*_R_* = 6.32 min, eluent E_2_. IR (KBr) ν (cm^−1^): 3339, 2873, 1606, 1513, 1483, 1338, 1229, 1200, 1033, 950, 819. ^1^H-NMR (500 MHz, CDCl_3_) δ (ppm): 9.28 (s, 1H, H-C(triazole)), 8.78 (s, 1H, H-C(2)), 7.81 (d, 2H, ^3^*J* = 8.8 Hz, 2 × H-C(Ar)), 6.85 (d, 2H, ^3^*J* = 8.6 Hz, 2 × H-C(Ar)), 6.75 (d, 2H, ^3^*J* = 8.8 Hz, 2 × H-C(Ar)), 6.68 (d, 2H, ^3^*J* = 8.6 Hz, 2 × H-C(Ar)), 4.89 (s, 2H, (-CH_2_-)), 4.55 (t, 2H, ^3^*J* = 5.0 Hz, (-CH_2_-)), 4.22 (t, 2H, ^3^*J* = 5.0 Hz, (-CH_2_-)), 4.10 (t, 2H, ^3^*J* = 4.7 Hz, (-CH_2_-)), 3.92 (t, 2H, ^3^*J* = 4.7 Hz, (-CH_2_-)), 3.06 (s, 6H, 2 × (-CH_3_)), 2.82 (s, 6H, 2 × (-CH_3_)). ^13^C-NMR (126 MHz, CDCl_3_) δ (ppm): 158.6, 156.8, 152.1, 151.3, 150.6, 146.0, 145.9, 142.7, 131.4, 124.2, 122.7, 115.8, 115.0, 114.9, 111.8, 69.3, 68.2, 64.7, 61.2, 48.2, 42.0, 40.2. HRMS (ESI) m/z: [M + H]^+^ Calculated for C_28_H_34_N_9_O_3_ 544.2779; Found 544.2805 (4.78 ppm).

#### 3.2.17. 2-{6-[4-({2-[4-(Dimethylamino)phenoxy]ethoxy}methyl)-1*H*-1,2,3-triazol-1-yl]-8-[4-(Dimethylamino)phenyl]-9*H*-purin-9-yl}ethyl Methanesulfonate (**31**)

To a solution of compound **30** (689 mg, 1.27 mmol, 1.0 eq.) in DCM (5 mL), Et_3_N (176 µL, ρ = 0.73 g/cm^3^, 1.27 mmol, 1.0 eq.) was added. The reaction mixture was cooled to 0 °C. MsCl (0.11 mL, ρ = 1.48 g/cm^3^, 1.46 mmol, 1.15 eq.) was slowly added dropwise to the reaction mixture, and it was left to stir for 1 h at 25 °C. Then, the reaction mixture was washed with H_2_O (2 × 10 mL) and brine (10 mL). The organic phase was dried over anhydrous Na_2_SO_4_, filtered, and poured in cold hexane (200 mL). The resulting precipitate was filtered, providing product **31** (yield: 772 mg, 98%) as a dark orange amorphous solid. HPLC: t*_R_* = 5.34 min, eluent E_2_. IR (KBr) ν (cm^−1^): 2929, 2868, 1608, 1514, 1477, 1339, 1221, 1170, 1038, 898, 810. ^1^H-NMR (500 MHz, CDCl_3_) δ (ppm): 9.34 (s, 1H, H-C(triazole)), 8.86 (s, 1H, H-C(2)), 7.76 (d, 2H, ^3^*J* = 8.4 Hz, 2 × H-C(Ar)), 6.86 (d, 2H, ^3^*J* = 8.8 Hz, 2 × H-C(Ar)), 6.82 (d, 2H, ^3^*J* = 8.8 Hz, 2 × H-C(Ar)), 6.77 (d, 2H, ^3^*J* = 8.4 Hz, 2 × H-C(Ar)), 4.89 (s, 2H, (-CH_2_-)), 4.84–4.77 (m, 2H, (-CH_2_-)), 4.74–4.68 (m, 2H, (-CH_2_-)), 4.15–4.07 (m, 2H, (-CH_2_-)), 3.95–4.89 (m, 2H, (-CH_2_-)), 3.06 (s, 6H, 2 × (-CH_3_)), 2.87 (s, 6H, 2 × (-CH_3_)), 2.84 (s, 3H, (-CH_3_)). ^13^C NMR (126 MHz, CDCl_3_) δ (ppm): 157.9, 156.8, 152.7, 152.2, 151.2, 145.8, 144.1, 143.1, 130.8, 124.4, 122.8, 116.1, 115.9, 114.6, 111.9, 69.1, 68.1, 65.6, 64.6, 43.8, 42.8, 40.2, 37.6. HRMS (ESI) m/z: [M + H]^+^ Calculated for C_29_H_36_N_9_O_5_S 622.2555; Found 625.2549 (0.94 ppm).

#### 3.2.18. 4-(6-(4-((2-(4-(Dimethylamino)phenoxy)ethoxy)methyl)-1*H*-1,2,3-triazol-1-yl)-9-(2-(4,7,10-trimethyl-1,4,7,10-tetraazacyclododecan-1-yl)ethyl)-9*H*-purin-8-yl)-*N*,*N*-Dimethylaniline (**1**)

A suspension of compound **31** (770 mg, 1.24 mmol, 1.0 eq.) and LiBr (215 mg, 2.48 mmol, 2.0 eq.) in THF (16 mL) was stirred in a pressure flask at 70 °C for 3 h. Then, the reaction mixture was evaporated, suspended in DCM (10 mL), and washed with water (2 × 10 mL) and brine (10 mL). The organic phase was dried over anhydrous Na_2_SO_4_, filtered, and evaporated. 1,4,7-Trimethyl-1,4,7,10-tetraazacyclododecane [75] (2.66 g, 12.4 mmol, 10 eq.) was added to the precipitate, and this mixture was dissolved in abs. MeCN (60 mL) and left to stir for 72 h at 25 °C. Silica gel column chromatography (DCM/EtOH, gradient 0% → 5%) provided by-product **32** (yield: 370 mg, 57%) as a yellow amorphous solid. Reverse phase silica gel column chromatography (MeOH/H_2_O, gradient 0% → 70%) with the following preparative HPLC column chromatography (that used TFA as an additive) was used to separate impurities from the product. Then, it was dissolved in DCM (10 mL), washed with sat. aqueous NaHCO_3_ solution (10 mL) and brine (10 mL), and evaporated. Next, it was dissolved in water (10 mL), filtered, and lyophilized, providing product **1** (yield: 56 mg, 6%) as a yellow amorphous solid. R_f_ = 0.1 (DCM/EtOH = 20:1). HPLC: t*_R_* = 4.38 min, eluent E_2_. IR (KBr) ν(cm^−1^): 2924, 2852, 1667, 1607, 1585, 1464, 1338, 1259, 1066, 1033, 796. ^1^H-NMR (500 MHz, CDCl_3_) δ 9.41 (ppm) (s, 1H, H-C(triazole)), 8.89 (s, 1H, H-C(2)), 7.83 (d, 2H, ^3^*J* = 8.8 Hz, 2 × H-C(Ar)), 6.86 (d, 2H, ^3^*J* = 9.0 Hz, 2 × H-C(Ar)), 6.80 (d, 2H, ^3^*J* = 8.8 Hz, 2 × H-C(Ar)), 6.67 (d, 2H, ^3^*J* = 9.0 Hz, 2 × H-C(Ar)), 4.92 (s, 2H, (-CH_2_-)), 4.59 (t, 2H, ^3^*J* = 6.8 Hz, (-CH_2_-)), 4.11 (t, 2H, ^3^*J* = 4.8 Hz, (-CH_2_-)), 3.92 (t, 2H, ^3^*J* = 4.8 Hz, (-CH_2_-)), 3.07 (s, 6H, 2 × (CH_3_)), 2.82 (s, 6H, 2 × (CH_3_)), 2.77 (t, 2H, ^3^*J* = 6.8 Hz, (-CH_2_-)), 2.65–2.61 (m, 4H, 2 × (-CH_2_-)), 2.32–2.26 (m, 12H, 6 × (-CH_2_-)), 2.16 (s, 3H, (-CH_3_)), 2.11 (s, 6H, 2 × (-CH_3_)). ^1^H-NMR (500 MHz, MeCN-d_3_) δ 9.27 (ppm) (s, 1H, H(1)), 8.84 (s, 1H, H(2)), 7.85 (d, 2H, ^3^*J* = 8.6 Hz, 2 × H(3)), 6.88 (d, 2H, ^3^*J* = 8.6 Hz, 2 × H(4)), 6.81 (d, 2H, ^3^*J* = 9.1 Hz, 2 × H(5)), 6.66 (d, 2H, ^3^*J* = 9.1 Hz, 2 × H(6)), 4.81 (s, 2H, 2 × H(7)), 4.61 (t, 2H, ^3^*J* = 6.6 Hz, 2 × H(8)), 4.07 (t, 2H, ^3^*J* = 4.6 Hz, 2 × H(9)), 3.86 (t, 2H, ^3^*J* = 4.6 Hz, 2 × H(10)), 3.05 (s, 6H, 6 × H(11)), 2.86–2.74 (m, 8H, 6 × H(12), 2 × H(13)), 2.58 – 2.51 (m, 4H, 4 × H(14)), 2.46 – 2.17 (m, 21H, 12 × H(15), 9 × H(16)). ^13^C NMR (126 MHz, CDCl_3_) δ (ppm): 157.8, 157.2, 152.1, 151.13, 151.03, 146.0, 145.8, 142.9, 130.7, 124.6, 122.7, 115.9, 115.8, 114.8, 111.9, 69.1, 68.2, 64.7, 56.4, 55.79, 55.75, 54.6, 53.4, 45.0, 44.4, 42.9, 41.8, 40.2. HRMS (ESI) m/z: [M + H]^+^ Calculated for C_39_H_58_N_13_O_2_ 740.4831; Found 740.4846 (2.03 ppm).

#### 3.2.19. 4-(6-(4-((2-(4-(Dimethylamino)phenoxy)ethoxy)methyl)-1*H*-1,2,3-triazol-1-yl)-9-vinyl-9*H*-purin-8-yl)-*N*,*N*-Dimethylaniline (**32**)

R_f_ = 0.61 (DCM/EtOH = 20:1). HPLC: t*_R_* = 6.21 min, eluent E_2_. IR (KBr) ν (cm^−1^): 2925, 2865, 1606, 1480, 1342, 1199, 1124, 1036, 1023, 946, 819. ^1^H NMR (500 MHz, CDCl_3_) δ 9.39 (s, 1H, H-C(triazole)), 8.95 (s, 1H, H-C(2)), 7.84 (d, 2H, ^3^*J* = 8.6 Hz, 2 × H-C(Ar)), 7.07 (dd, 1H, ^3^*J* = 15.8, 8.9 Hz, (-CH_a_)), 6.86 (d, 2H, ^3^*J* = 8.6 Hz, 2 × H-C(Ar)), 6.81 (d, 2H, ^3^*J* = 8.6 Hz, 2 × H-C(Ar)), 6.68 (d, 2H, ^3^*J* = 8.6 Hz, 2 × H-C(Ar)), 6.43 (d, 1H, ^2^*J* = 15.8 Hz, (-CH_b_)), 5.51 (d, 1H, ^3^*J* = 8.9 Hz, (-CH_c_)), 4.93 (s, 2H, (-CH_2_-)), 4.12 (t, 2H, ^3^*J* = 4.8 Hz, (-CH_2_-)), 3.94 (t, 2H, ^3^*J* = 4.8 Hz, (-CH_2_-)), 3.09 (s, 6H, 2 × (-CH_3_)), 2.83 (s, 6H, 2 × (-CH_3_)). ^13^C NMR (126 MHz, CDCl_3_) δ (ppm): 156.8, 156.2, 152.3, 151.5, 151.1, 146.0, 145.9, 143.2, 131.6, 128.4, 124.5, 123.4, 115.9, 114.8, 114.7, 111.7, 110.9, 69.2, 68.3, 64.7, 41.8, 40.2. HRMS (ESI) m/z: [M + H]^+^ Calculated for C_28_H_32_N_9_O_2_ 526.2673; Found 526.2688 (2.85 ppm).

## 4. Conclusions

We have developed a new synthetic approach toward the proposed purine–cyclen conjugate using the sequence of modifications, which includes Mitsunobu, iodination, Stille, S_N_Ar, CuAAC, and alkylation reactions as main transformations. The optimal sequence of derivatization (positions: *N*9 → *C*8 → *C*6 → *N*9) was found as the result of several less successful attempts toward the target compound. Purine–cyclen conjugate was obtained in nine steps, starting from commercially available 6-chloropurine. As the main by-product, the *N*9-vinyl group containing purine–triazole conjugate was obtained, which was formed via the E2 type reaction mechanism between purine alkyl bromide derivative and modified cyclen moiety. Preliminary NMR and UV titration experiments revealed that target purine–cyclen conjugate easily complexes copper(II) ions in the cyclen ring and between triazole *N*2 and purine *N*7 positions. Photophysical and photocatalytic properties of the developed purine–cyclen derivatives will be studied in the future and will be reported elsewhere.

## Data Availability

The original contributions presented in this study are included in the article/Supplementary Materials. Further inquiries can be directed to the corresponding authors.

## Supplementary Materials

The following supporting information can be downloaded at: https://www.mdpi.com/article/10.3390/molecules30071612/s1, ^1^H- and ^13^C-NMR spectra of compounds **1**, **3**, **11**, **13**, **15**, **22**–**32**; NMR titration procedure and stacked spectra for the titration experiments of compound **1**.
