# Supplementary material for: Synthesis of Purine-1,4,7,10-Tetraazacyclododecane Conjugate and Its Complexation Modes with Copper(II)
Source: Molecules. 2025 Apr 4;30(7):1612. doi: 10.3390/molecules30071612 (PMC11990475; doi:10.3390/molecules30071612)
Supplement: Supplementary file 1 [file molecules-30-01612-s001.zip › molecules-3536665-supplementary.pdf]

Supplementary Materials

Synthesis of purine-1,4,7,10-tetraazacyclododecane conjugate and its complexation modes with copper(II)

Aleksejs Burcevs [1], Gediminas Jonusauskas [2], Irina Novosjolova [1,*] and Māris Turks [1,*]

[1] Institute of Chemistry and Chemical Technology, Faculty of Natural Sciences and Technology, Riga Technical University, P. Valdena Str. 3, LV-1048 Riga, Latvia; aleksejs.burcevs@rtu.lv (A.B.)
[2] Laboratoire Ondes et Matière d'Aquitaine, Bordeaux University, UMR CNRS 5798, 351 Cours de la Libération, Talence, 33405, France; gediminas.jonusauskas@u-bordeaux.fr (G.J.)
* Correspondence: maris.turks@rtu.lv; irina.novosjolova@rtu.lv

Table of Contents

1. General information.. S3

2. Spectral data of compounds **1**, **3**, **11**, **13**, **15**, **22–32**.. S4

3. NMR titration experiment.. S21

1. General information

Analytical thin-layer chromatography (TLC) was performed on Merck 60 Å silica gel F_{254} plates. Column chromatography was performed on Merck 40–60 µm 60 Å silica gel or *Licroprep C18 (25–40* µm) silica gel.
NMR spectra were recorded on Bruker Avance 500 (Bruker, Billerica, MA, USA) spectrometer. ^{1}H-NMR spectra were recorded at 500 MHz with internal references from nondeuterated solvents (δ = 7.26 for $CDCl_3$, δ = 2.50 for DMSO-d_6, δ = 1.94 for MeCN-d_3). ^{13}C-NMR spectra were recorded at 125.7 MHz with internal references from nondeuterated solvents (δ = 77.16 for $CDCl_3$, δ = 39.52 for DMSO-d_6). Coupling constants are reported in Hz, chemical shifts of signals are given in ppm, and standard abbreviations were used for multiplicity assignments.
The IR spectra were recorded in hexachlorobutadiene (4000–2000 cm^{-1}) with an FTIR PerkinElmer Spectrum 100 (PerkinElmer, Waltham, MA, USA) or using Thermo Scientific Nicolet™ iSTM50 (Thermo Fisher, Waltham, MA, USA) spectrometer in the Attenuated Total Reflectance (ATR) mode. Spectra were obtained over a range of wavenumbers from 400 cm^{-1} to 4000 cm^{-1} co-adding 64 scans at 4 cm^{-1} resolution. Before every measurement, a background spectrum was taken and deducted from the sample spectrum.
HPLC analyses were performed using Agilent Technologies 1200 Series system (Agilent Technologies, Santa Clara, CA, USA) equipped with XBridge *C18* column, 4.6×150 mm, particle size 3.5 µm, with flow rate of 1 mL/min, using 0.1% TFA/H_2O and MeCN for the mobile phase. The wavelength of detection was 260 nm. Eluent A – 0.1% TFA aqueous solution with 5% v/v MeCN, eluent B – MeCN. Eluent E_1 – gradient 30–95% B 5 min, 95% B 5 min, 95–30% B 2 min.
HRMS analysis was performed on an Agilent 1290 Infinity series UPLC system (Agilent Technologies), connected to Agilent 6230 TOF LC/MS mass spectrometer (Agilent Technologies); column Extend C18 RRHD 2.1 × 50 mm, particle size 1.8 µm. Eluents: formic acid in CH_3CN (0.1 %) and aq. 0.1 formic acid.

2. Spectral data of compounds 1, 3, 11, 13, 15, 22–32

***N*,*N*-Dimethyl-4-(2-(prop-2-yn-1-yloxy)ethoxy)aniline (3)**

^{1}H-NMR (500 MHz, $CDCl_3$) spectrum of compound 3:

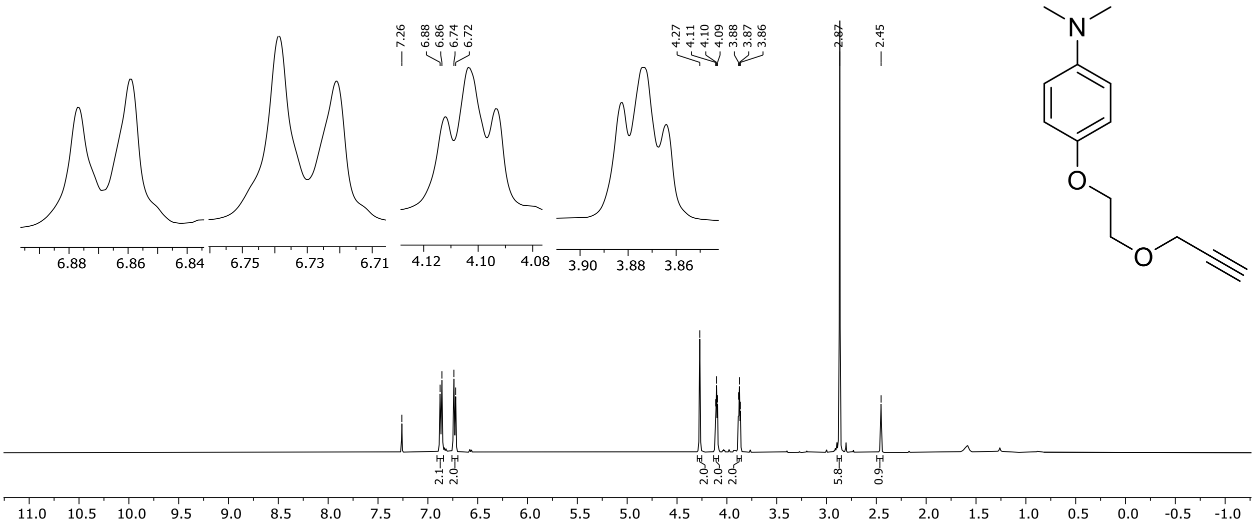

^{13}C-NMR (126 MHz, $CDCl_3$) spectrum of compound 3:

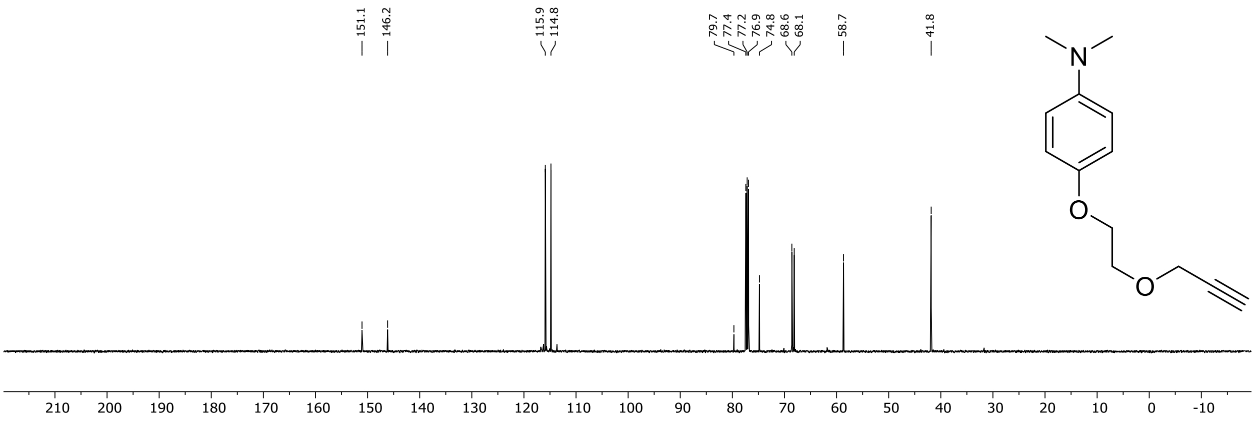

6-Azido-9-(tetrahydro-2*H*-pyran-2-yl)-9*H*-purine (11)

^{1}H-NMR (500 MHz, DMSO-d$_6$) spectrum of compound 11:

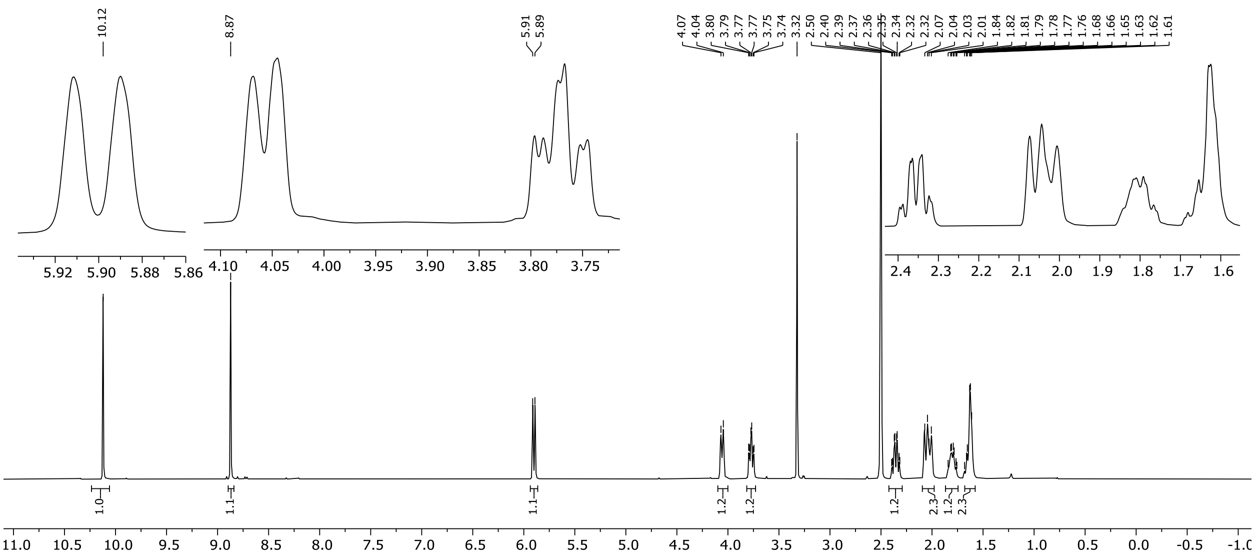

^{13}C-NMR (126 MHz, DMSO-d$_6$) spectrum of compound 11:

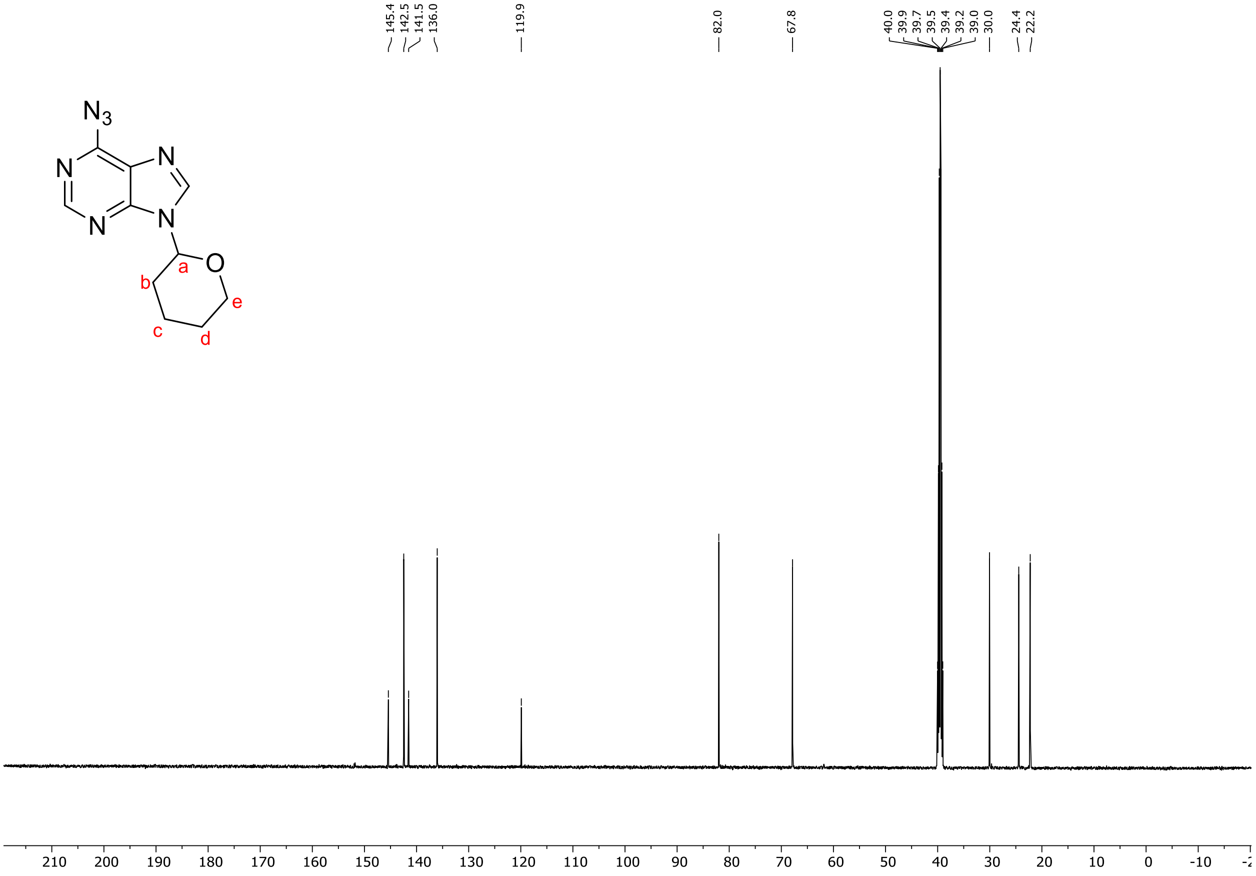

2-({1-[9-(Tetrahydro-2*H*-pyran-2-yl)-9*H*-purin-6-yl]-1*H*-1,2,3-triazol-4-yl}methoxy)ethan-1-ol (13)

^{1}H-NMR (500 MHz, $CDCl_3$) spectrum of compound 13:

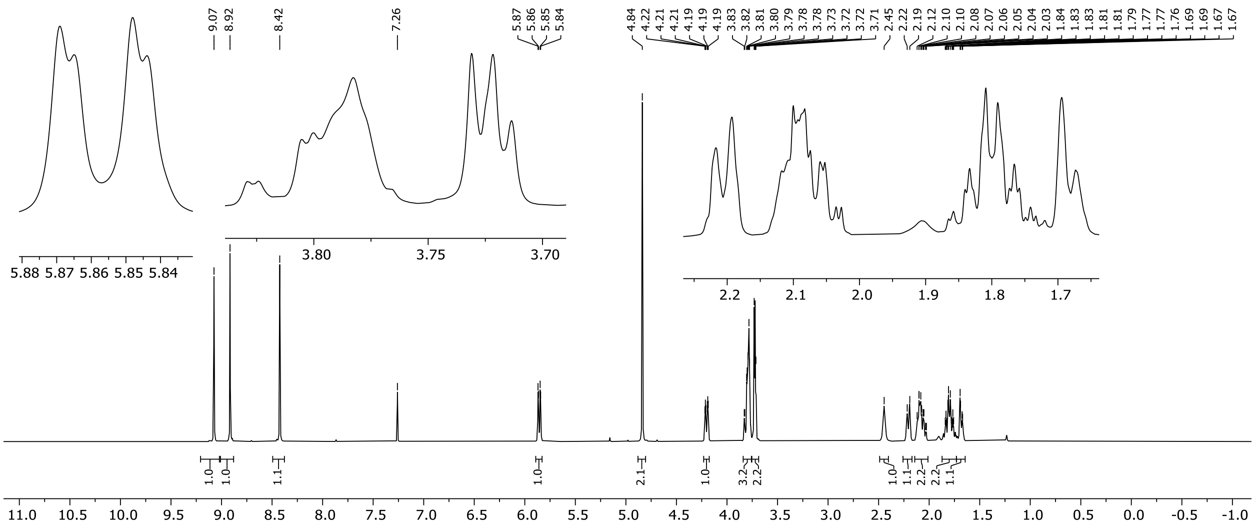

^{13}C-NMR (126 MHz, $CDCl_3$) spectrum of compound 13:

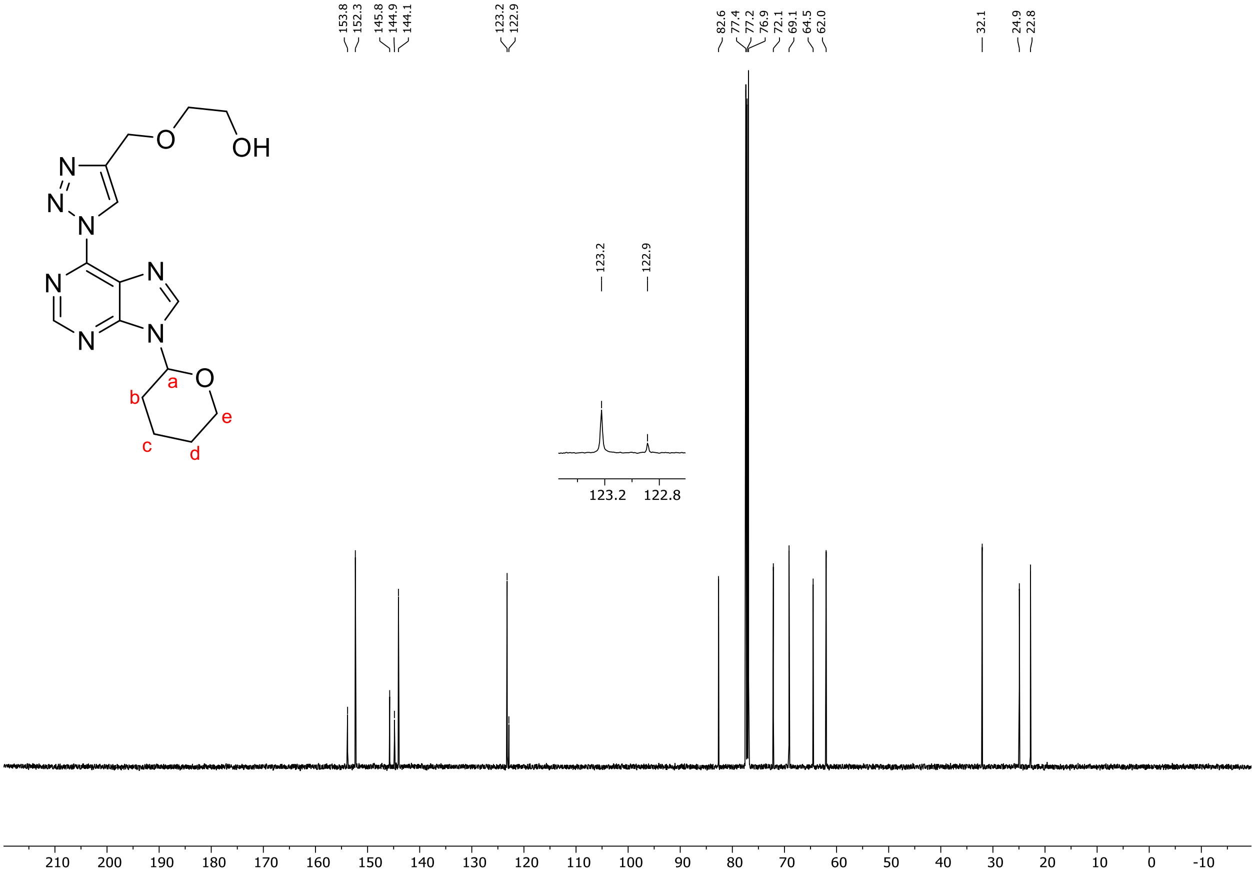

_N,N_-Dimethyl-4-[2-({1-[9-(tetrahydro-2_H_-pyran-2-yl)-9_H_-purin-6-yl]-1_H_-1,2,3-triazol-4-yl}methoxy)ethoxy]aniline (15)

^{1}H-NMR (500 MHz, $CDCl_3$) spectrum of compound 15:

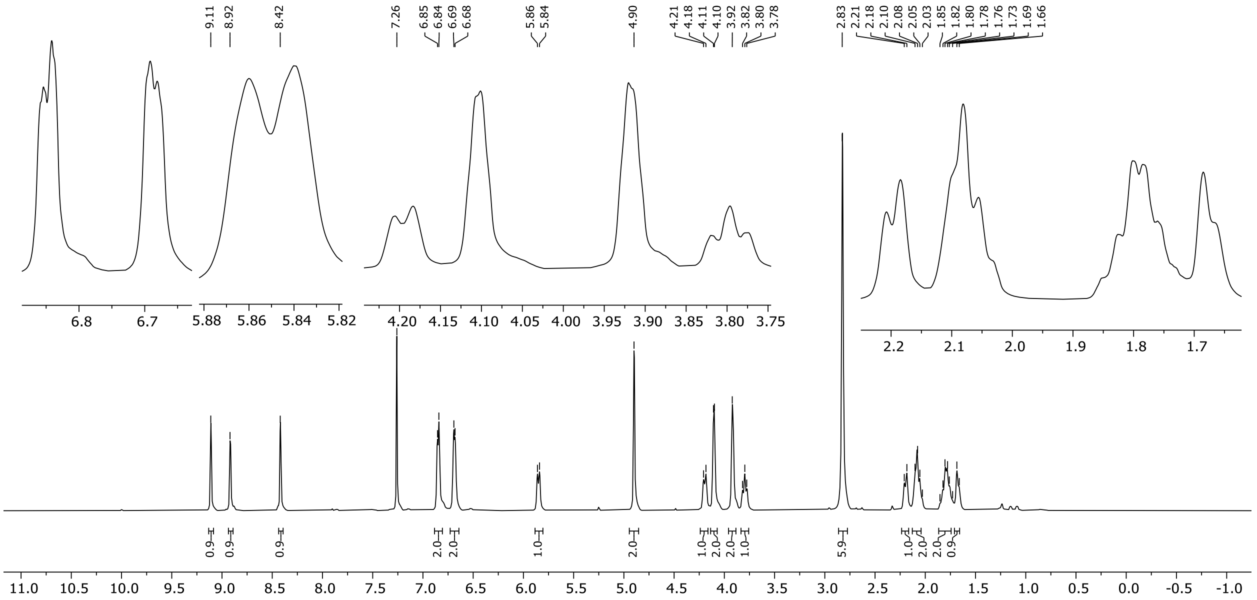

^{13}C-NMR (126 MHz, $CDCl_3$) spectrum of compound 15:

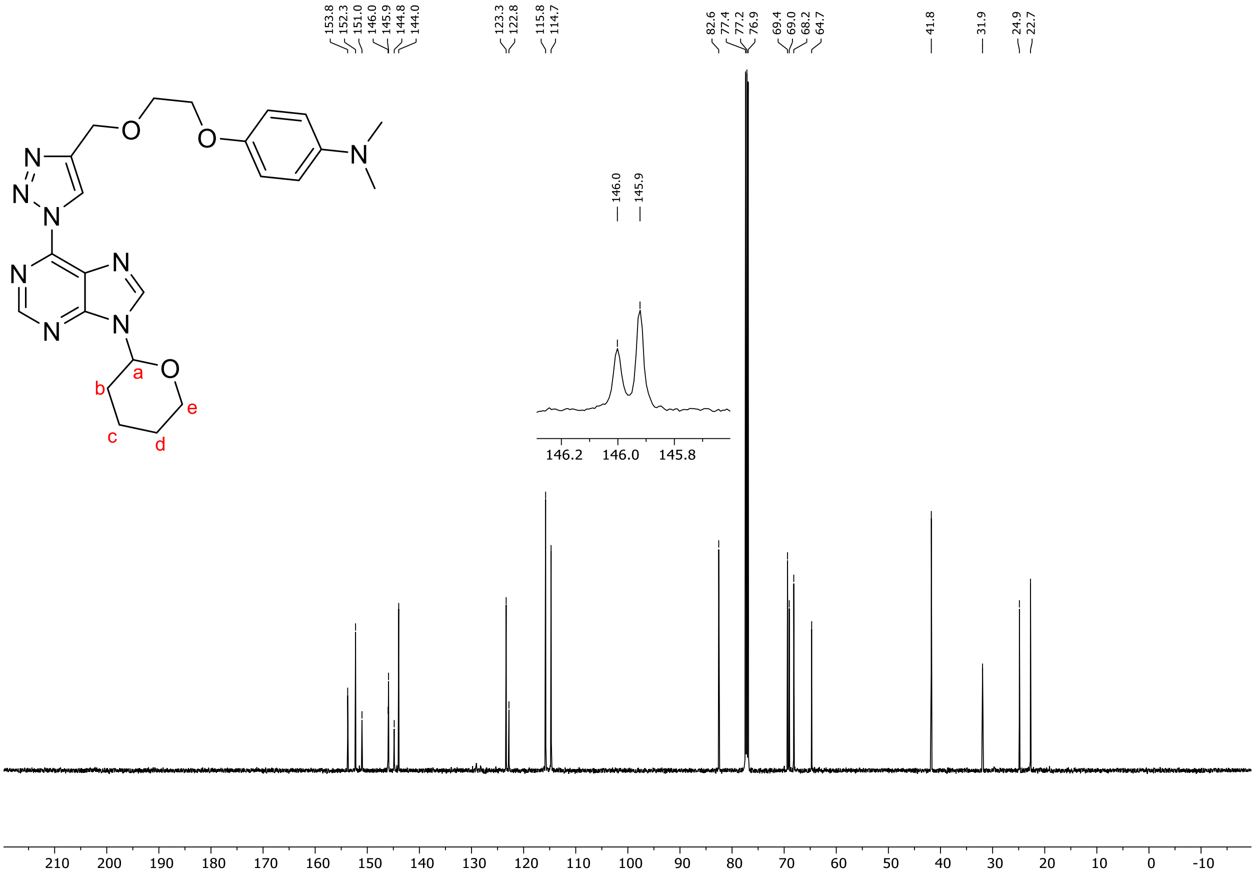

6-Chloro-9-{2-[(tetrahydro-2*H*-pyran-2-yl)oxy]ethyl}-9*H*-purine (22)

^{1}H-NMR (500 MHz, $CDCl_3$) spectrum of compound 22:

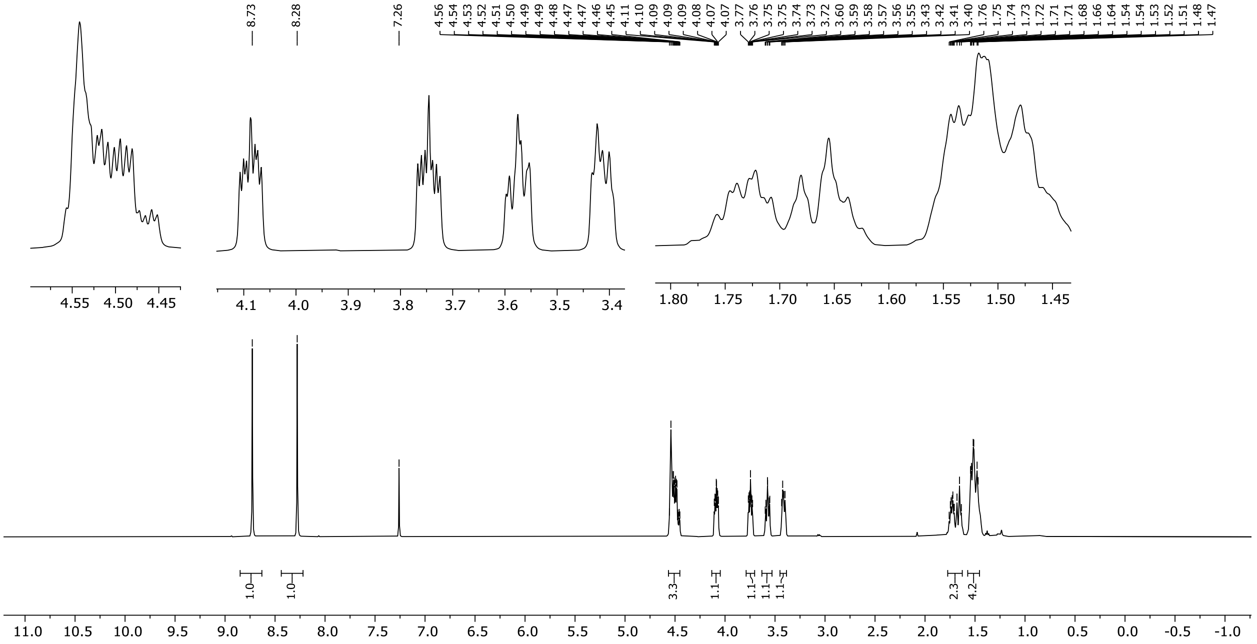

^{13}C-NMR (126 MHz, $CDCl_3$) spectrum of compound 22:

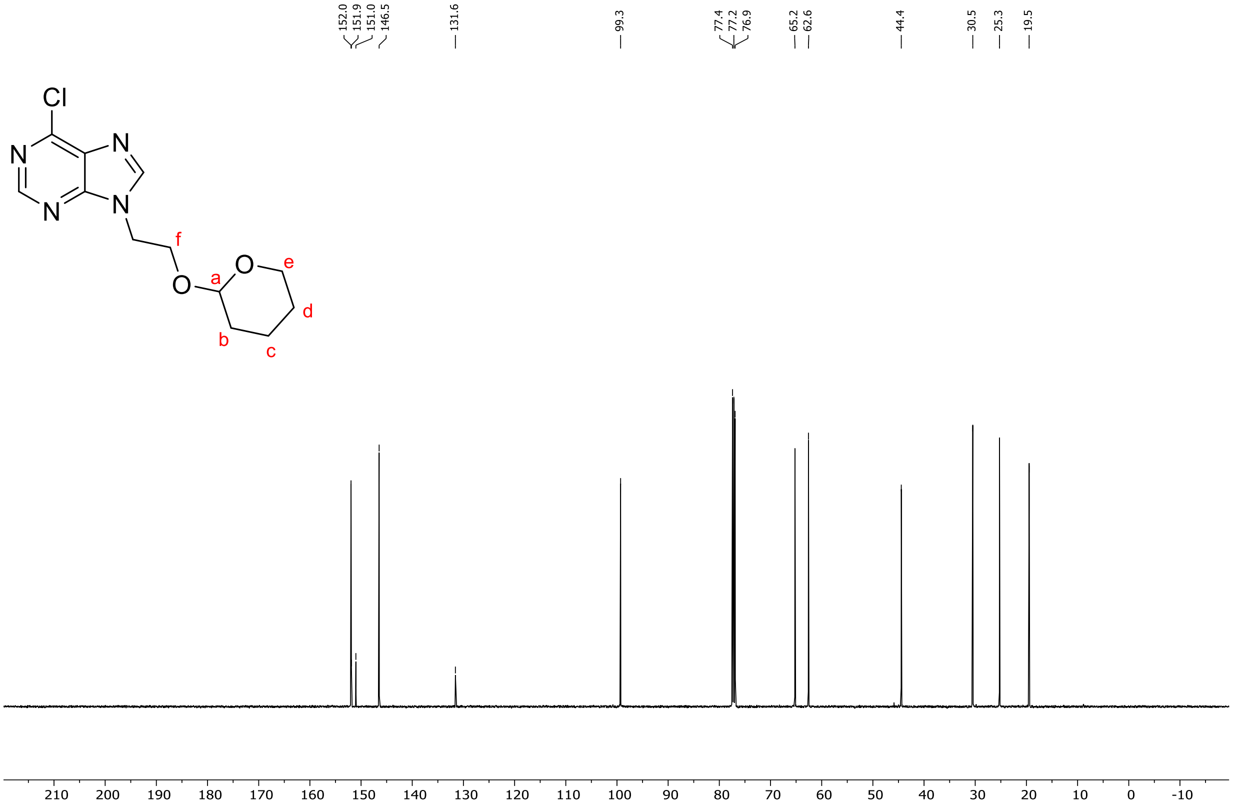

6-Azido-9-{2-[(tetrahydro-2*H*-pyran-2-yl)oxy]ethyl}-9*H*-purine (23)

^{1}H-NMR (500 MHz, DMSO-d$_6$) spectrum of compound 23:

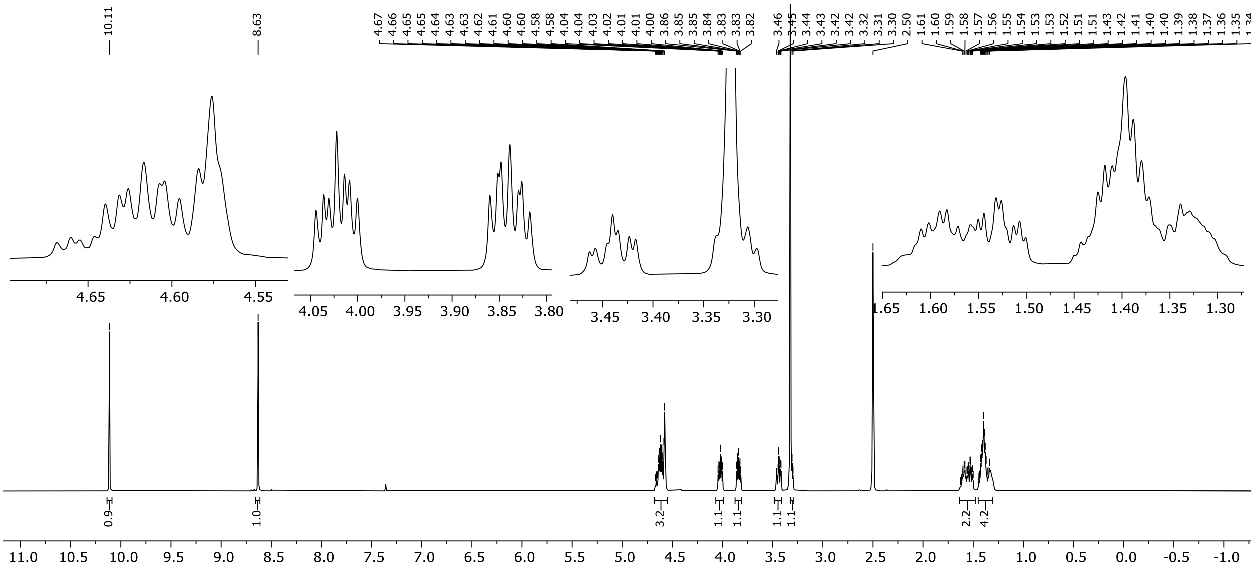

^{13}C-NMR (126 MHz, DMSO-d$_6$) spectrum of compound 23:

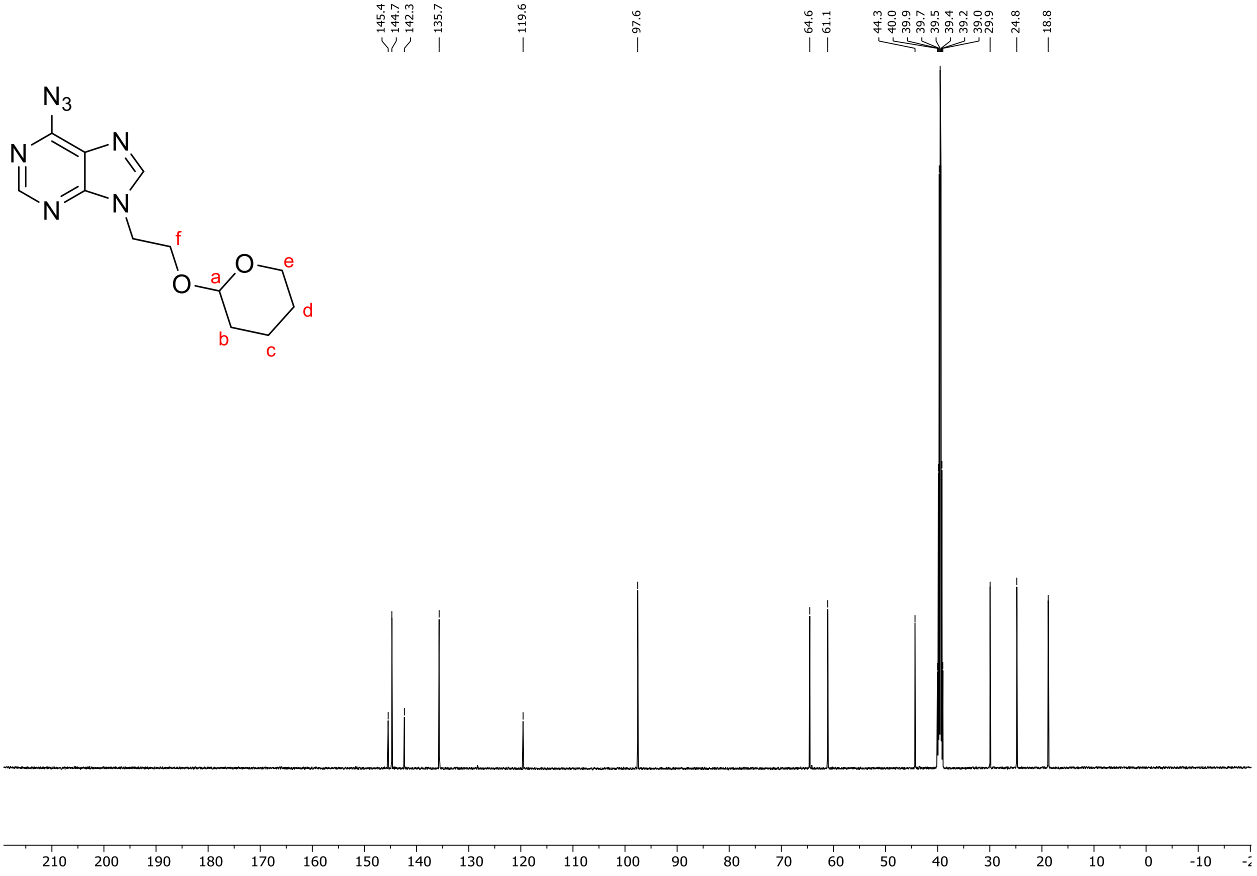

***N*,*N*-Dimethyl-4-(2-{[1-(9-{2-[(tetrahydro-2*H*-pyran-2-yl)oxy]ethyl}-9*H*-purin-6-yl)-1*H*-1,2,3-triazol-4-yl]methoxy}ethoxy)aniline (24)**

^{1}H-NMR (500 MHz, $CDCl_3$) spectrum of compound 24:

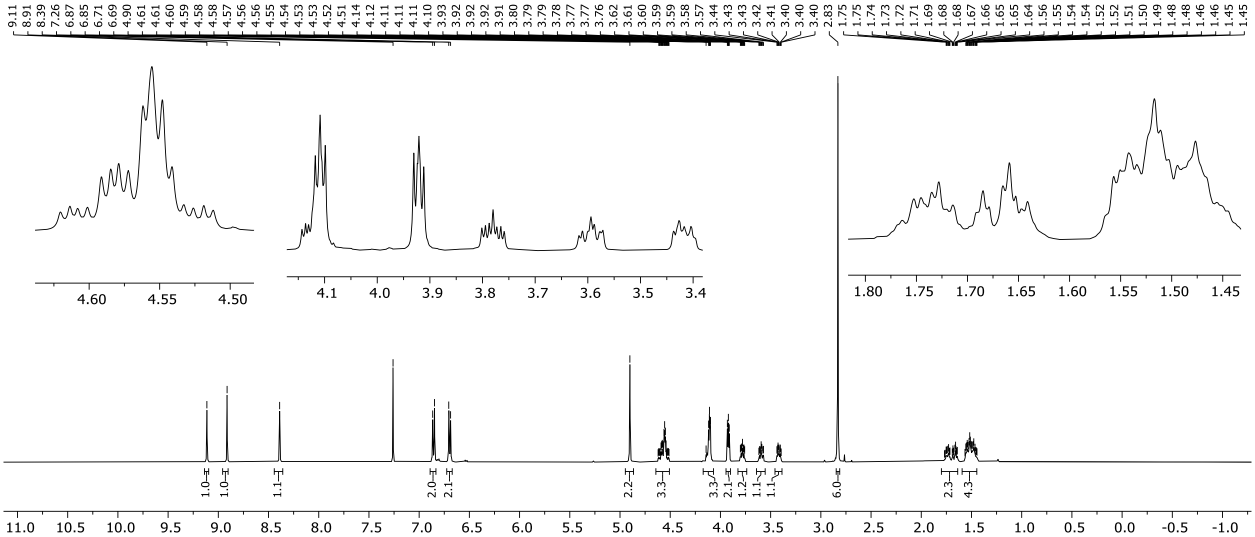

^{13}C-NMR (126 MHz, $CDCl_3$) spectrum of compound 24:

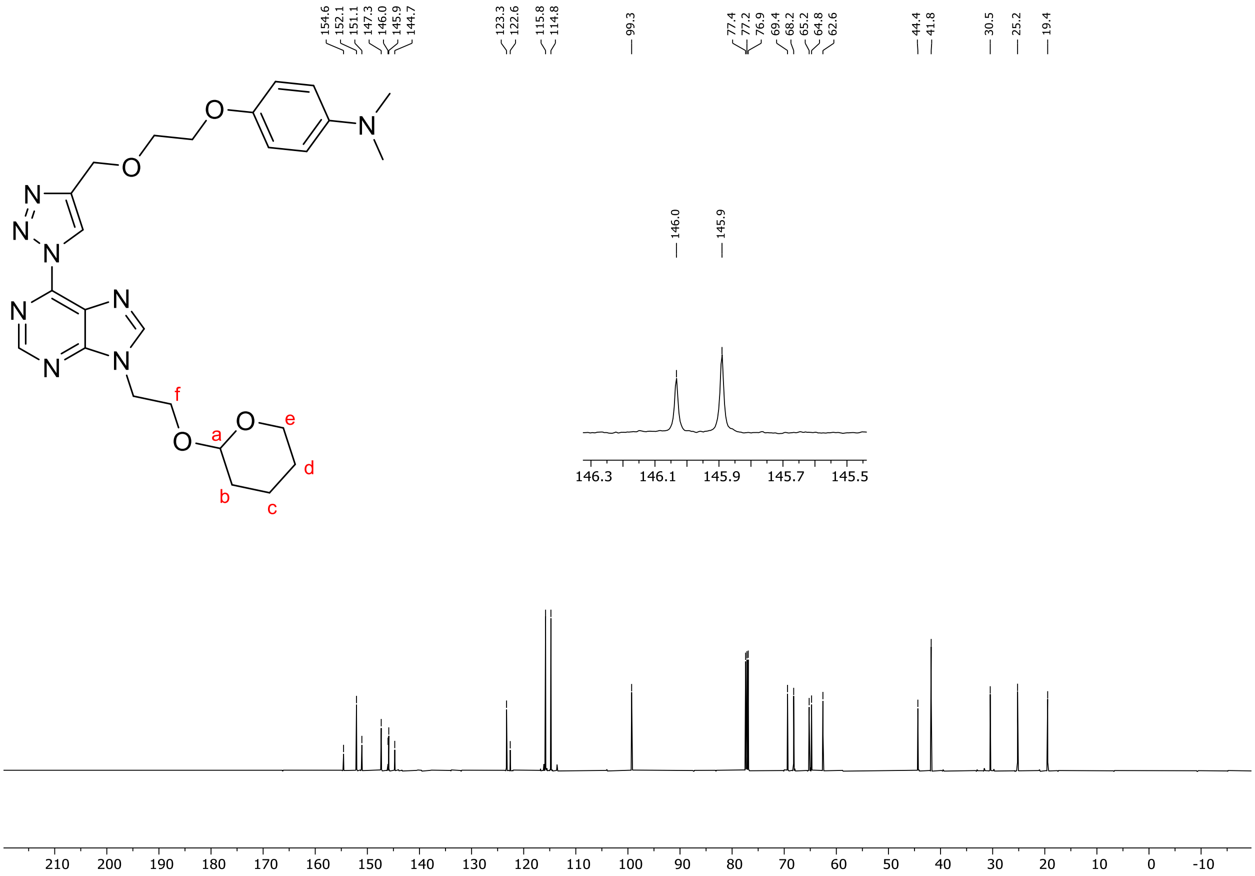

2-Bromo-*N*,*N*-dimethyl-4-{2-[(1-(9-{2-[(tetrahydro-2*H*-pyran-2-yl)oxy]ethyl}-9*H*-purin-6-yl)-1*H*-1,2,3-triazol-4-yl)methoxy]ethoxy}aniline (25)

^{1}H-NMR (500 MHz, $CDCl_3$) spectrum of compound 25:

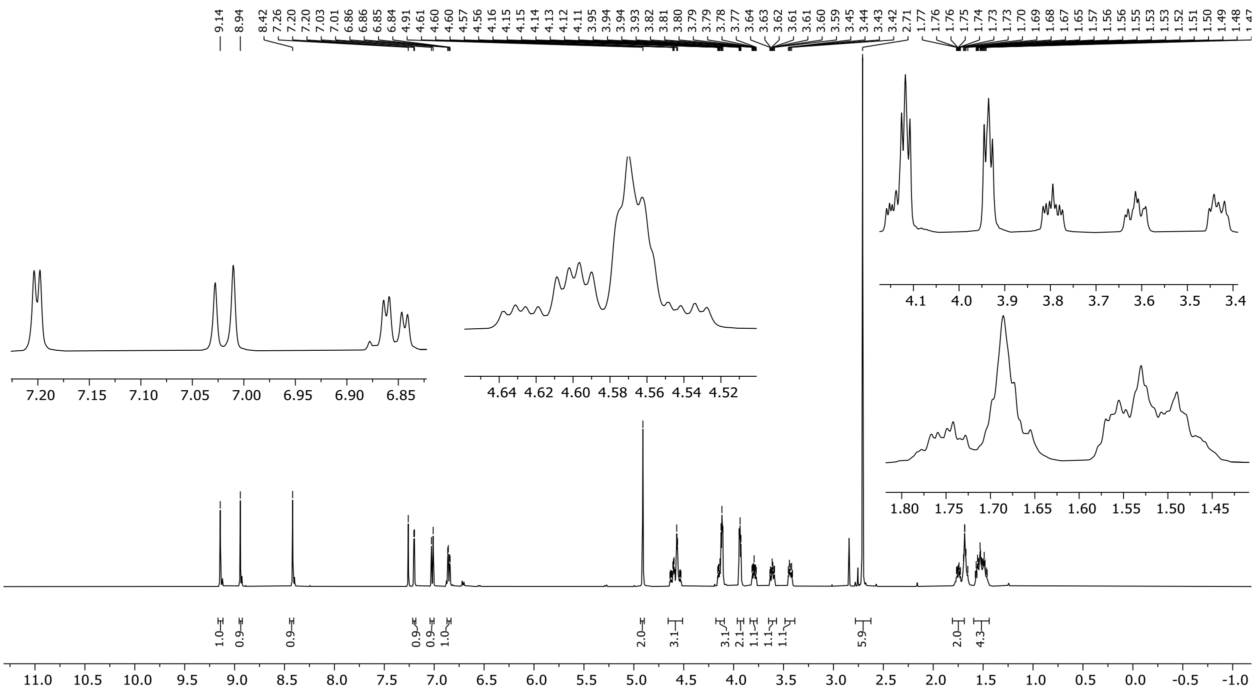

^{13}C-NMR (126 MHz, $CDCl_3$) spectrum of compound 25:

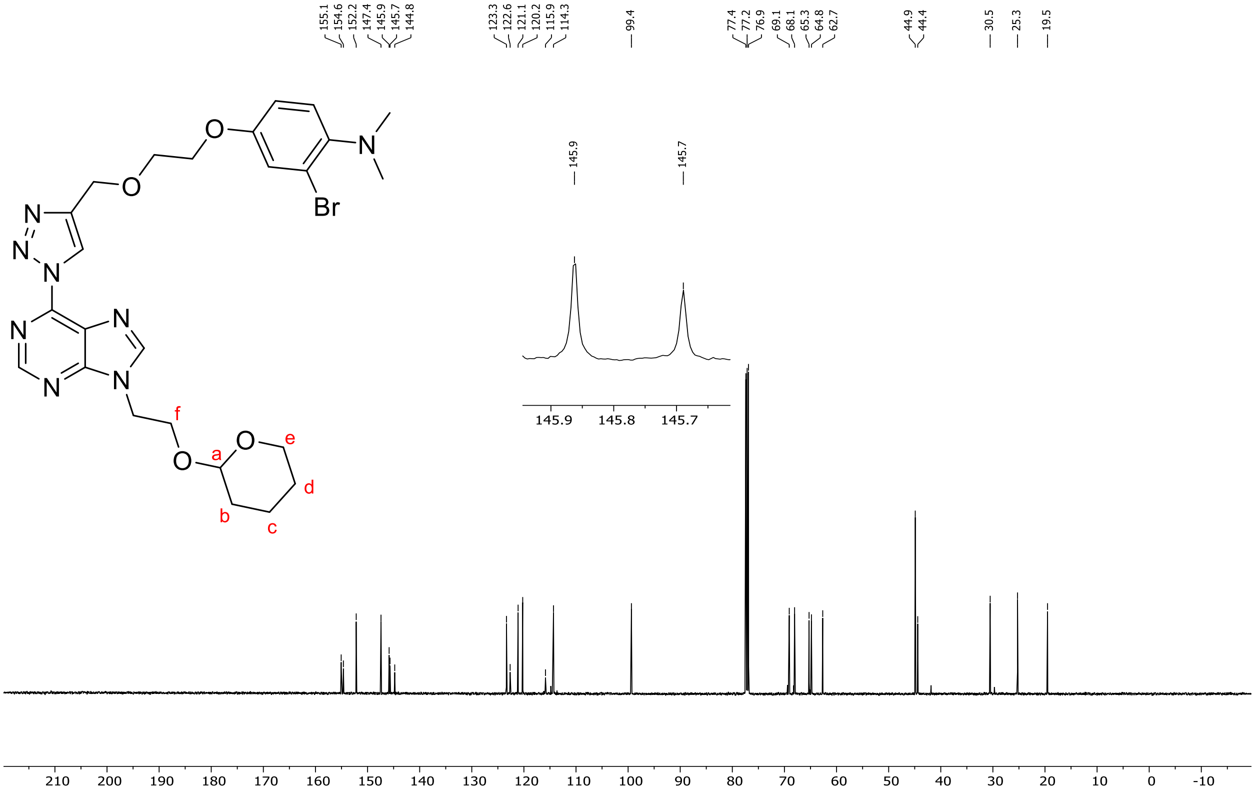

6-Chloro-8-iodo-9-{2-[(tetrahydro-2*H*-pyran-2-yl)oxy]ethyl}-9*H*-purine (26)

^{1}H-NMR (500 MHz, $CDCl_3$) spectrum of compound 26:

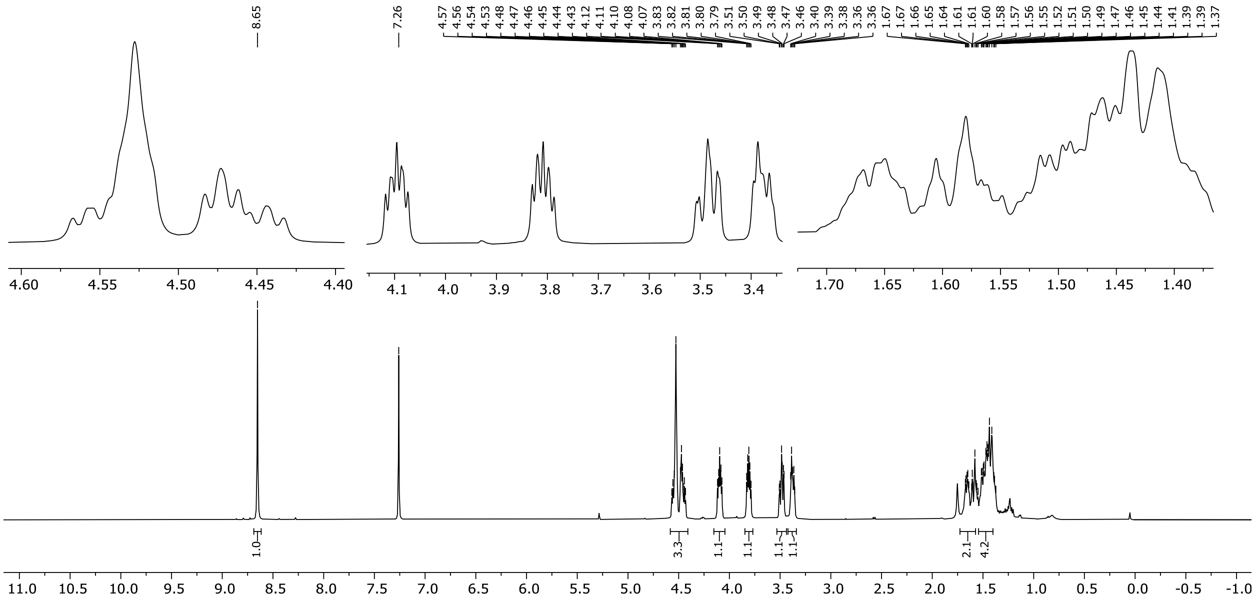

^{13}C-NMR (126 MHz, $CDCl_3$) spectrum of compound 26:

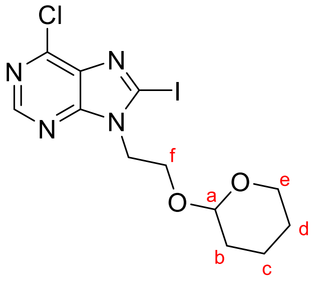

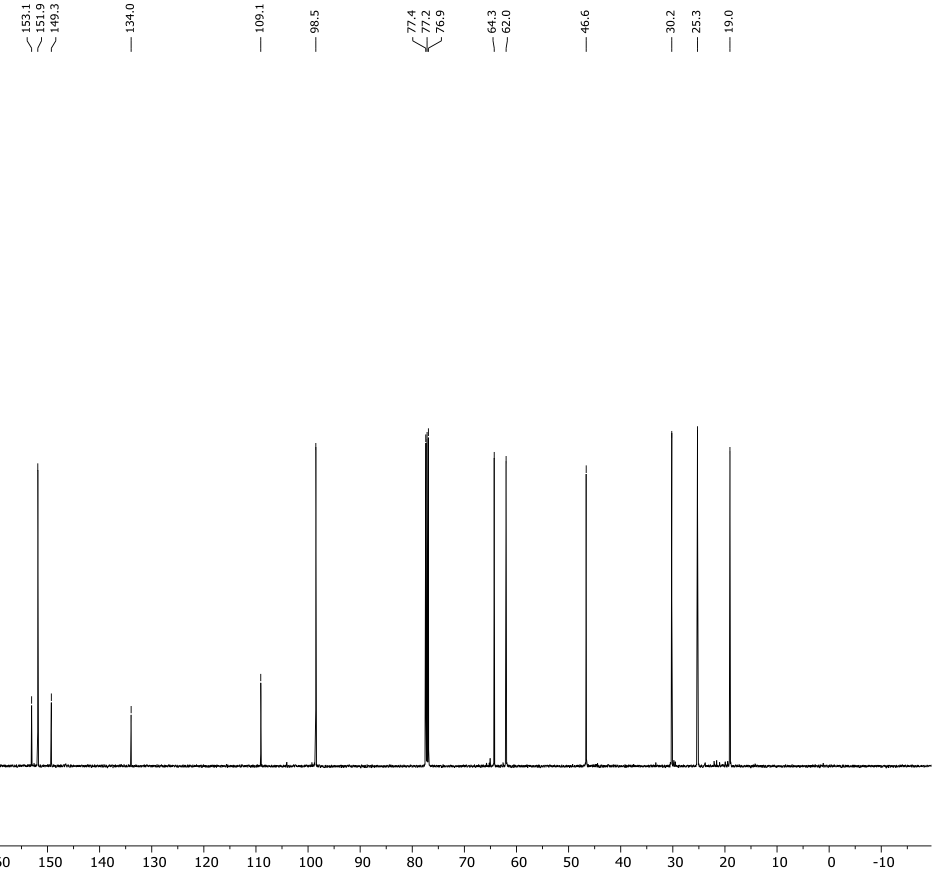

4-(6-Chloro-9-{2-[(tetrahydro-2*H*-pyran-2-yl)oxy]ethyl}-9*H*-purin-8-yl)-*N*,*N*-dimethylaniline (27)

^{1}H-NMR (500 MHz, CDCl$_3$) spectrum of compound 27:

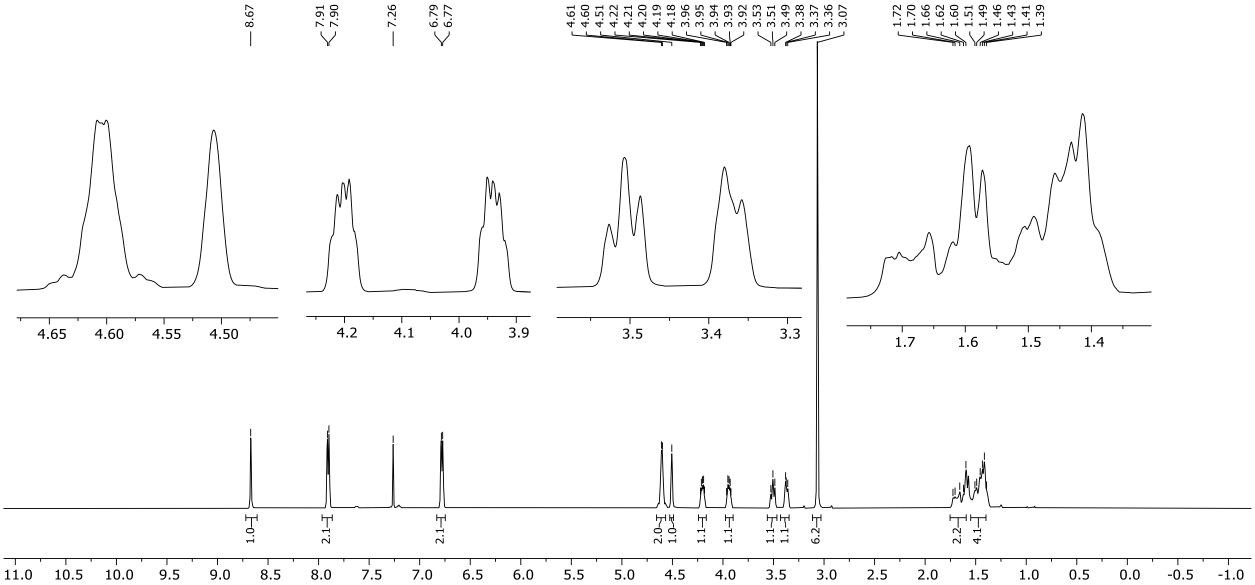

^{13}C-NMR (126 MHz, CDCl$_3$) spectrum of compound 27:

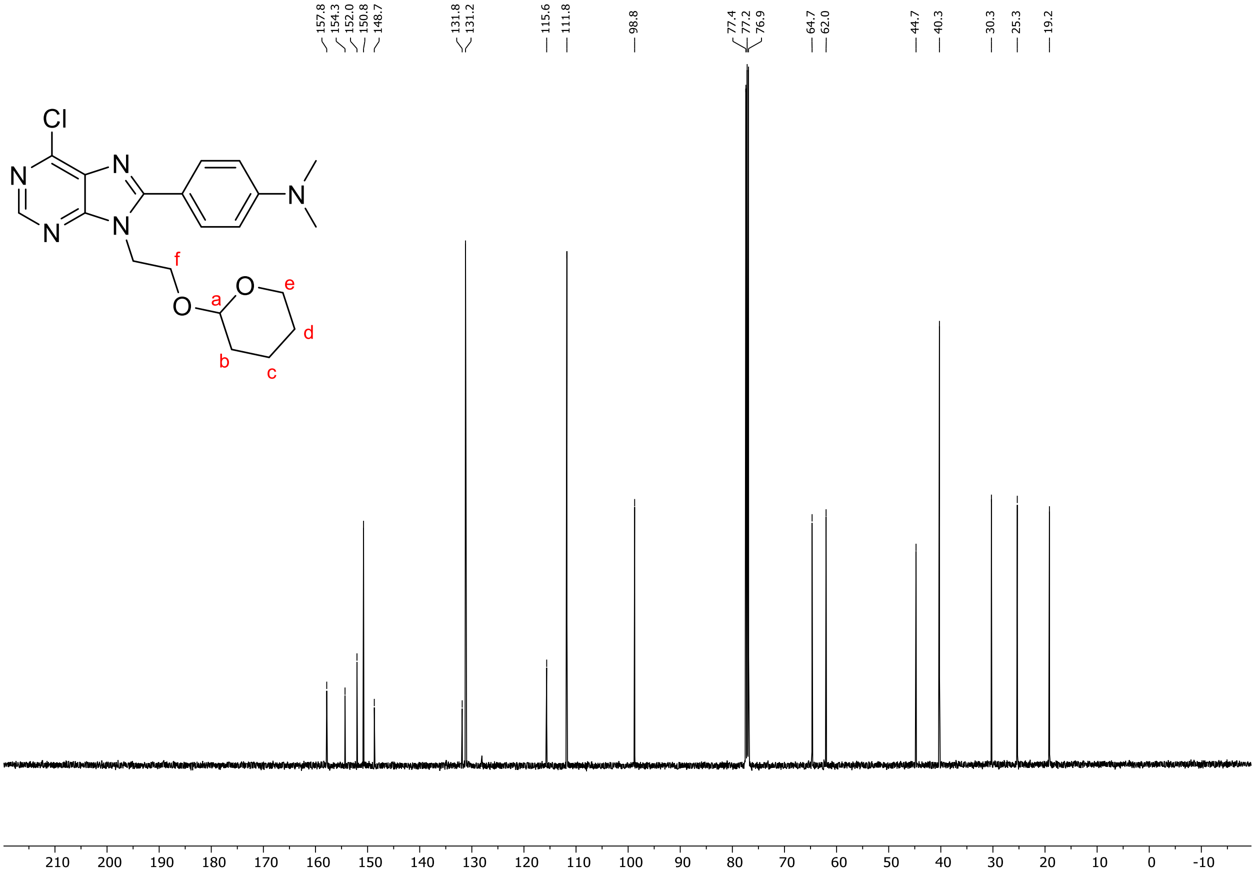

4-(6-Azido-9-{2-[(tetrahydro-2*H*-pyran-2-yl)oxy]ethyl}-9*H*-purin-8-yl)-*N*,*N*-dimethylaniline (28)

^{1}H-NMR (500 MHz, DMSO-d$_6$) spectrum of compound 28:

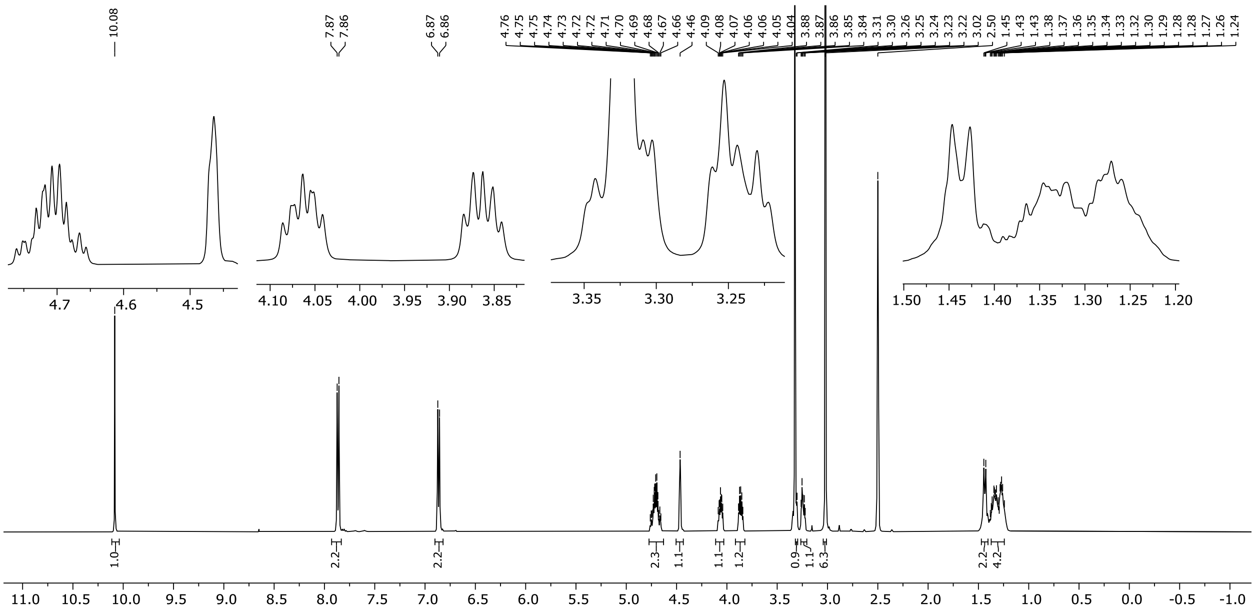

^{13}C-NMR (126 MHz, DMSO-d$_6$) spectrum of compound 28:

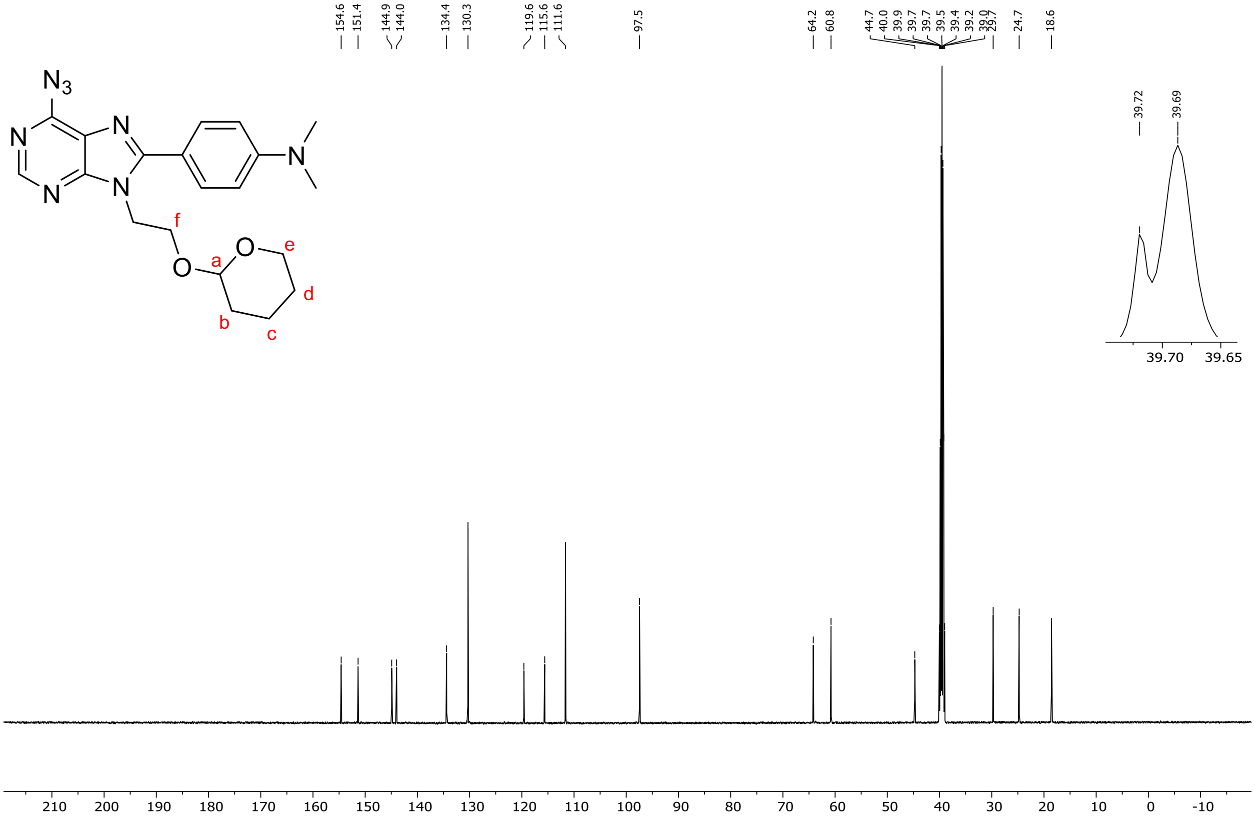

4-[6-(4-({2-[4-(Dimethylamino)phenoxy]ethoxy)methyl}-1*H*-1,2,3-triazol-1-yl)-9-{2-[(tetrahydro-2*H*-pyran-2-yl)oxy]ethyl}-9*H*-purin-8-yl]-*N*,*N*-dimethylaniline (29)

^{1}H-NMR (500 MHz, $CDCl_3$) spectrum of compound 29:

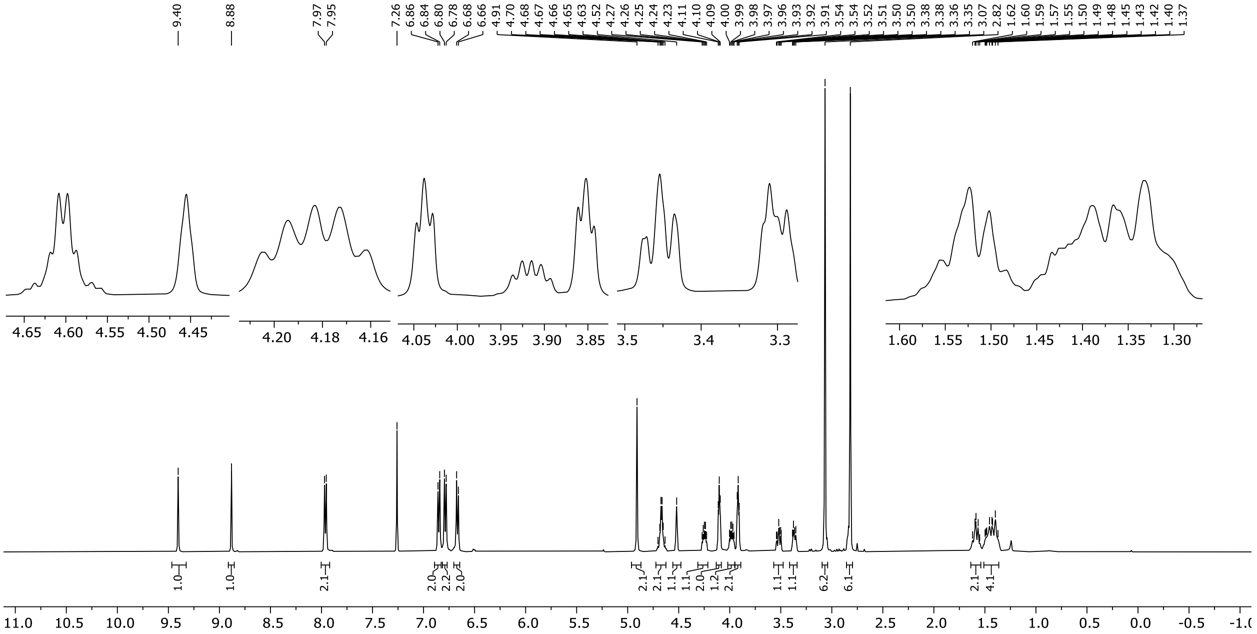

^{13}C-NMR (126 MHz, $CDCl_3$) spectrum of compound 29:

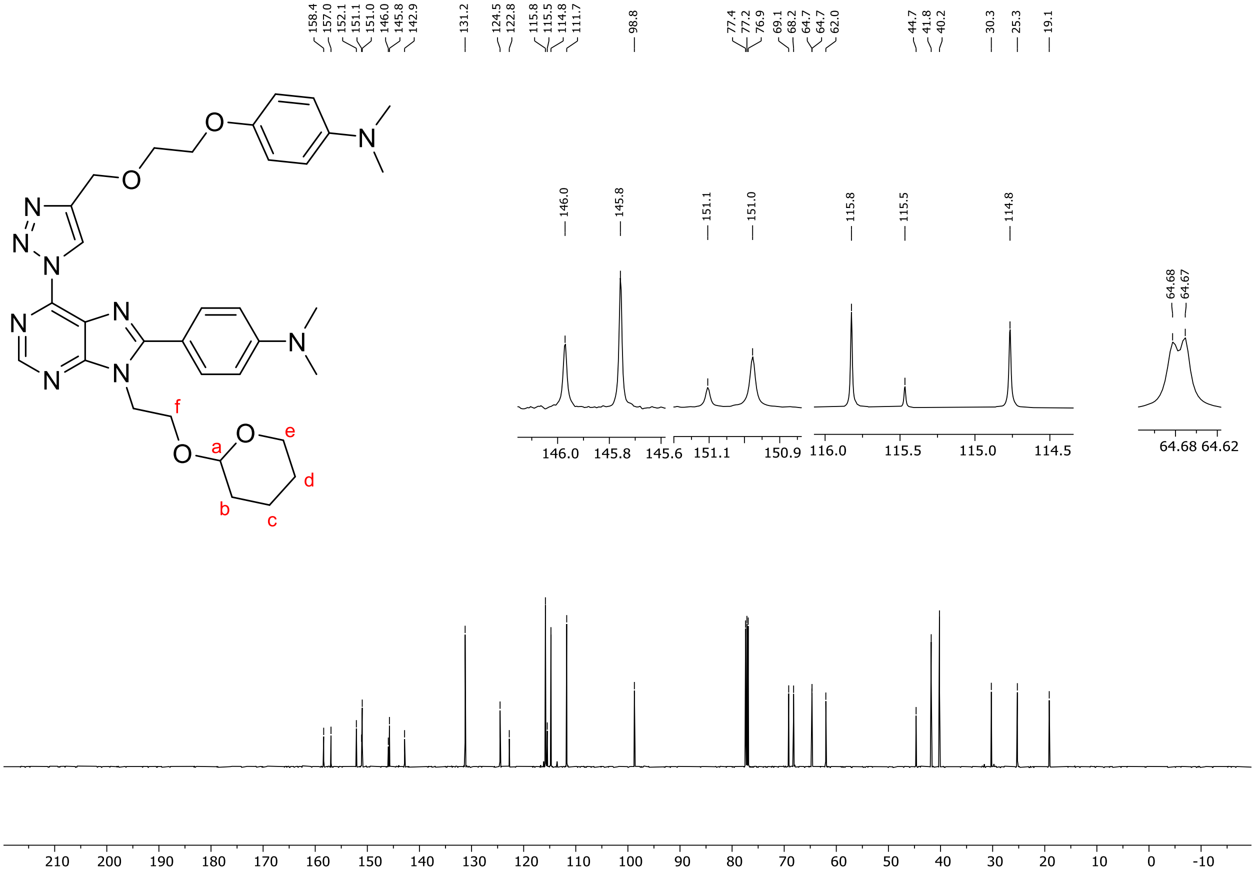

2-{6-[4-({2-[4-(Dimethylamino)phenoxy]ethoxy}methyl)-1*H*-1,2,3-triazol-1-yl]-8-[4-(dimethylamino)phenyl]-9*H*-purin-9-yl}ethan-1-ol (30)

^{1}H-NMR (500 MHz, $CDCl_3$) spectrum of compound 30:

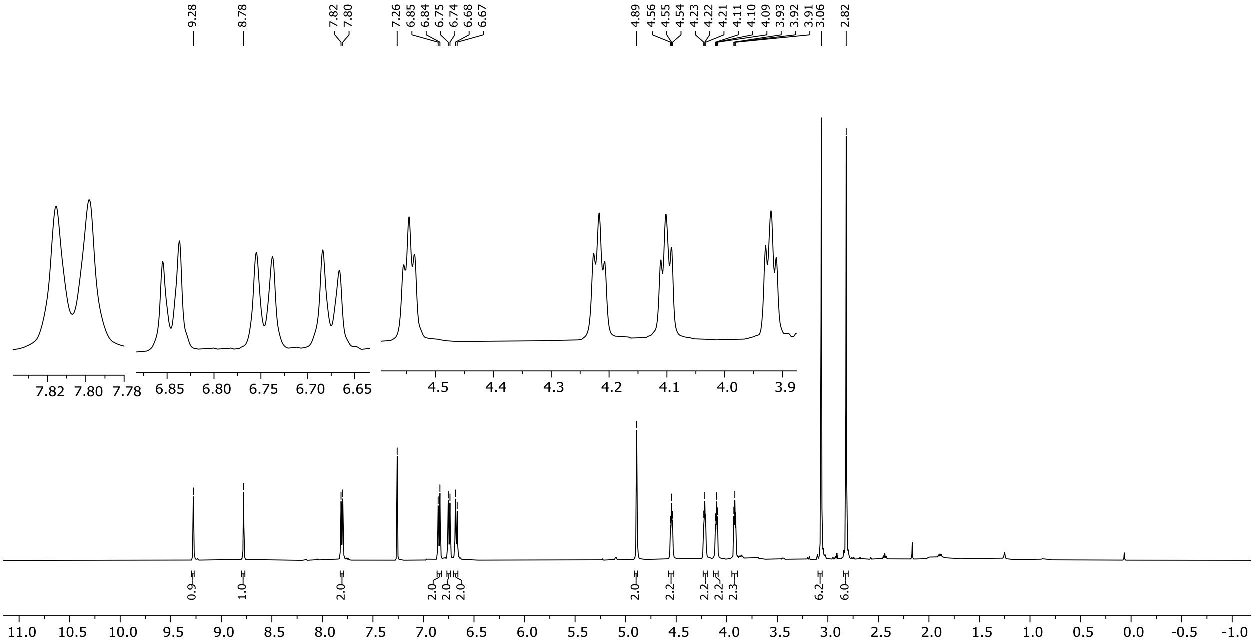

^{13}C-NMR (126 MHz, $CDCl_3$) spectrum of compound 30:

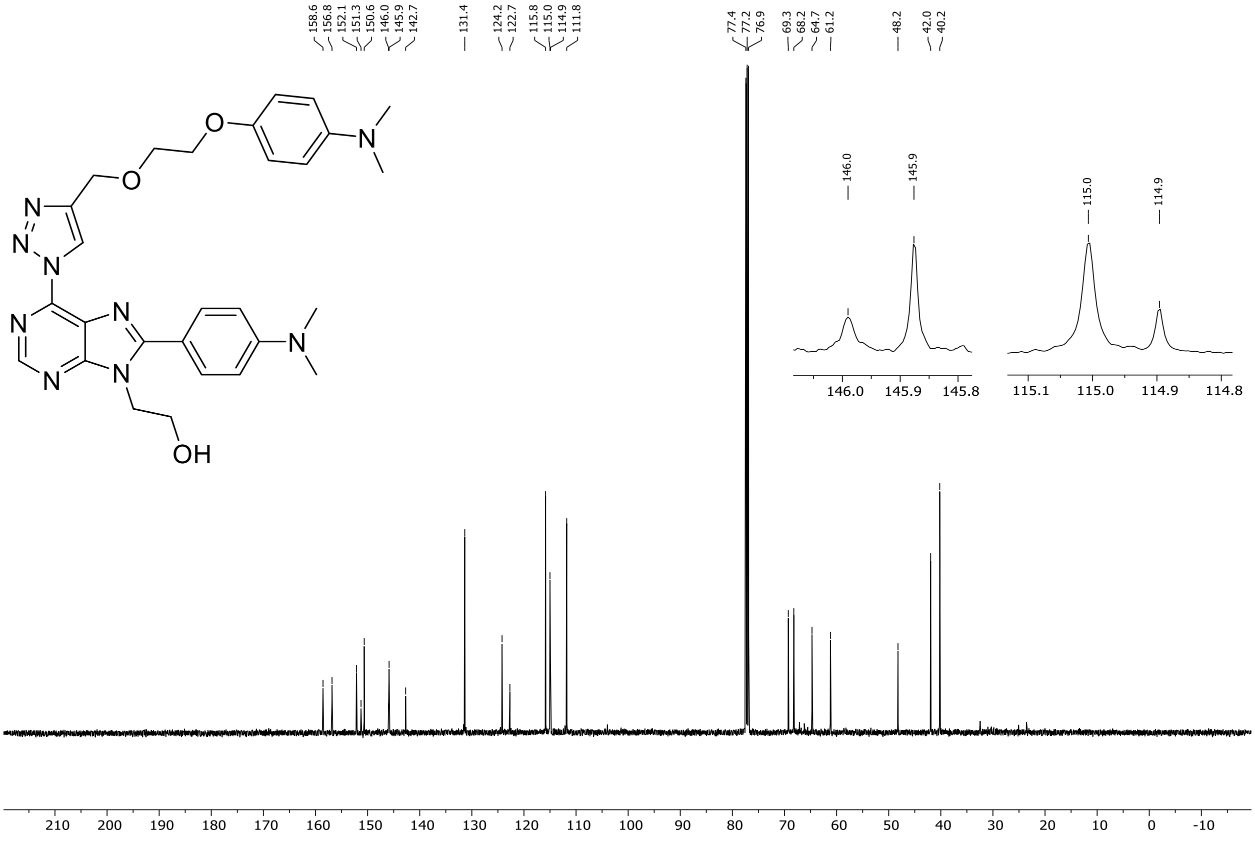

2-{6-[4-({2-[4-(Dimethylamino)phenoxy]ethoxy}methyl)-1*H*-1,2,3-triazol-1-yl]-8-[4-(dimethylamino)phenyl]-9*H*-purin-9-yl}ethyl methanesulfonate (31)

^{1}H-NMR (500 MHz, $CDCl_3$) spectrum of compound 31:

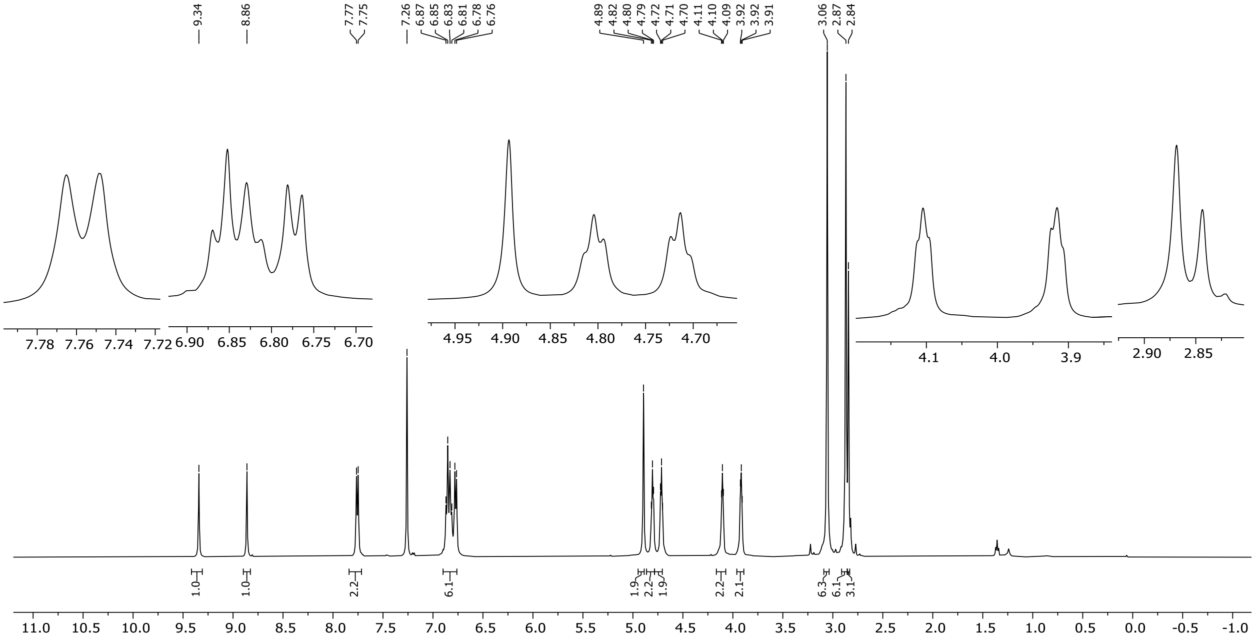

^{13}C-NMR (126 MHz, $CDCl_3$) spectrum of compound 31:

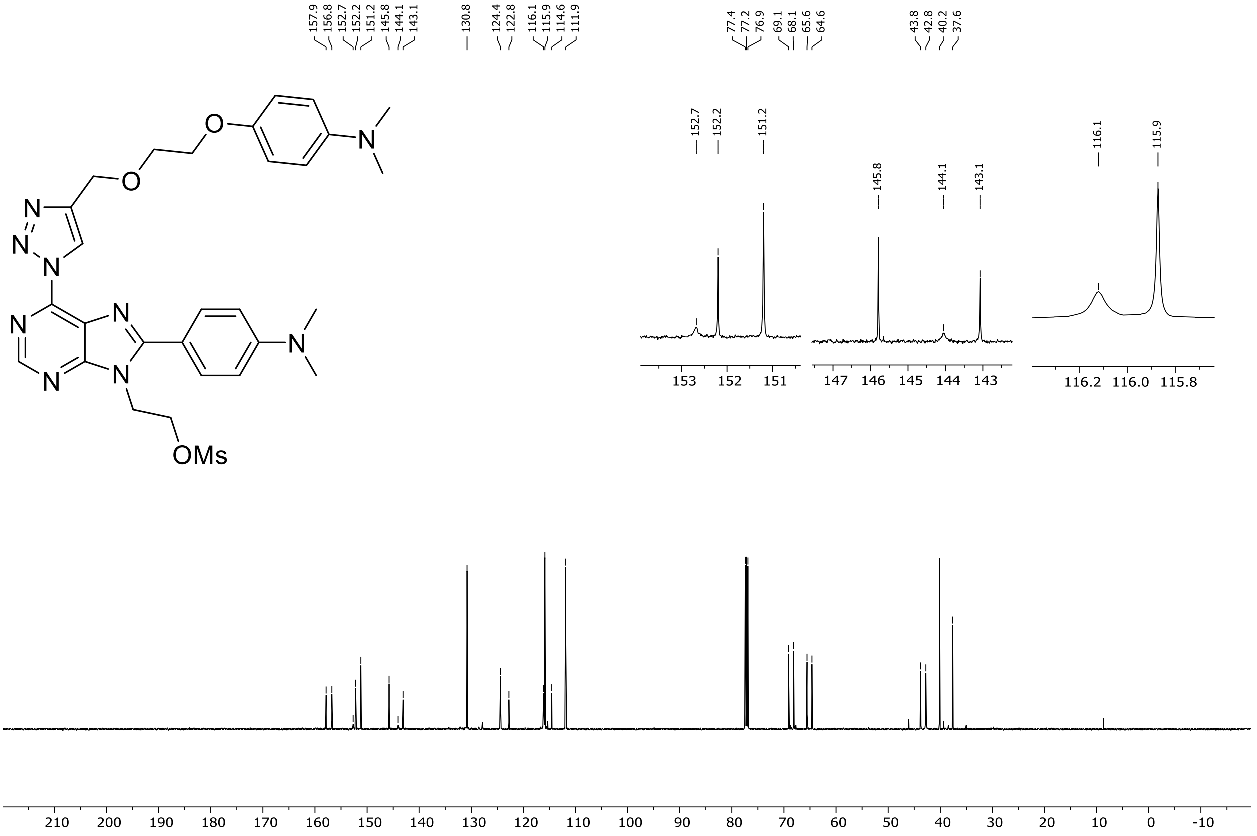

4-(6-(4-((2-(4-(Dimethylamino)phenoxy)ethoxy)methyl)-1*H*-1,2,3-triazol-1-yl)-9-(2-(4,7,10-trimethyl-1,4,7,10-tetraazacyclododecan-1-yl)ethyl)-9*H*-purin-8-yl)-*N*,*N*-dimethylaniline (1)

^{1}H-NMR (500 MHz, CDCl$_3$) spectrum of compound 1:

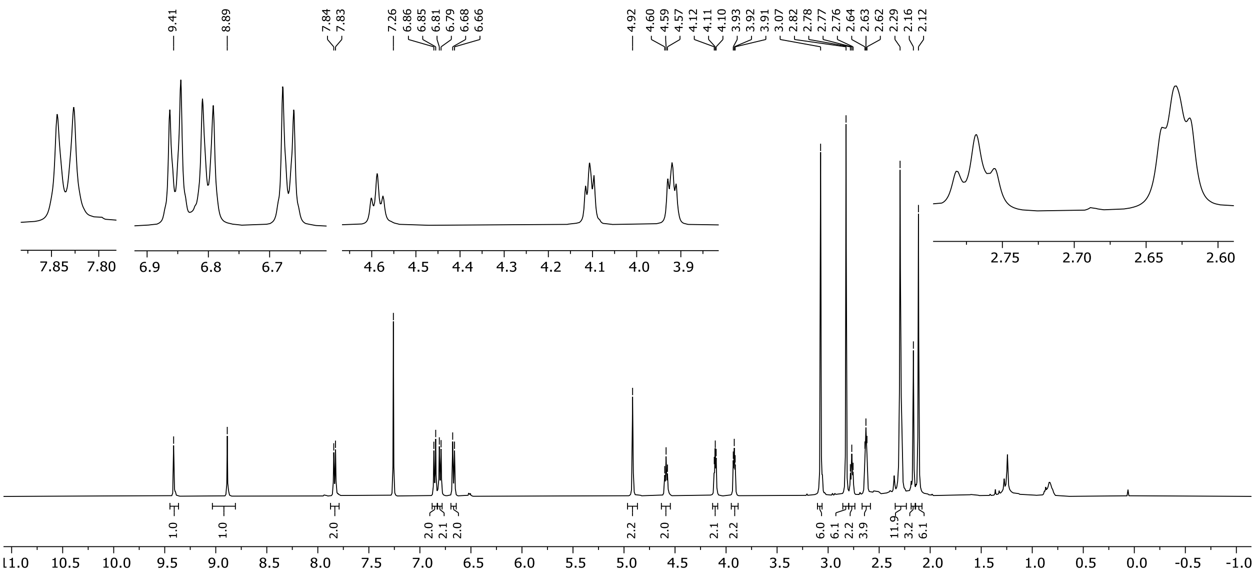

^{13}C-NMR (126 MHz, CDCl$_3$) spectrum of compound 1:

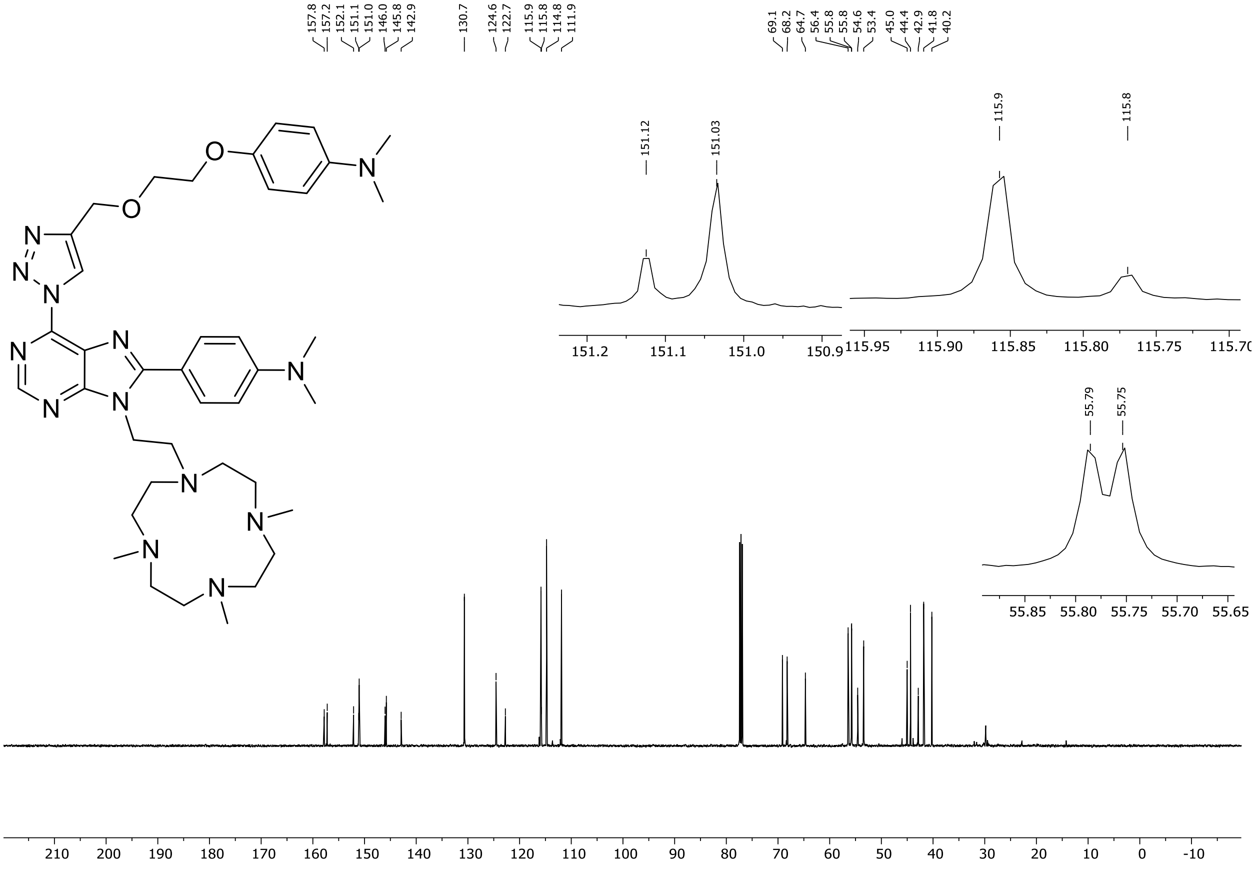

^{1}H-NMR (500 MHz, MeCN-d_3) spectrum of compound 1:

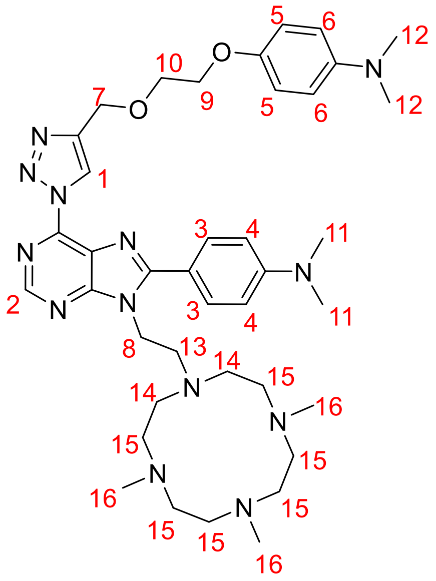

^{1}H-NMR (500 MHz, MeCN-d_3) δ 9.27 (ppm) (s, 1H, H(1)), 8.84 (s, 1H, H(2)), 7.85 (d, 2H, ^3J = 8.6 Hz, 2×H(3)), 6.88 (d, 2H, ^3J = 8.6 Hz, 2×H(4)), 6.81 (d, 2H, ^3J = 9.1 Hz, 2×H(5)), 6.66 (d, 2H, ^3J = 9.1 Hz, 2×H(6)), 4.81 (s, 2H, 2×H(7)), 4.61 (t, 2H, ^3J = 6.6 Hz, 2×H(8)), 4.07 (t, 2H, ^3J = 4.6 Hz, 2×H(9)), 3.86 (t, 2H, ^3J = 4.6 Hz, 2×H(10)), 3.05 (s, 6H, 6×H(11)), 2.86 – 2.74 (m, 8H, 6×H(12), 2×H(13)), 2.58 – 2.51 (m, 4H, 4×H(14)), 2.46 – 2.17 (m, 21H, 12×H(15), 9×H(16)).

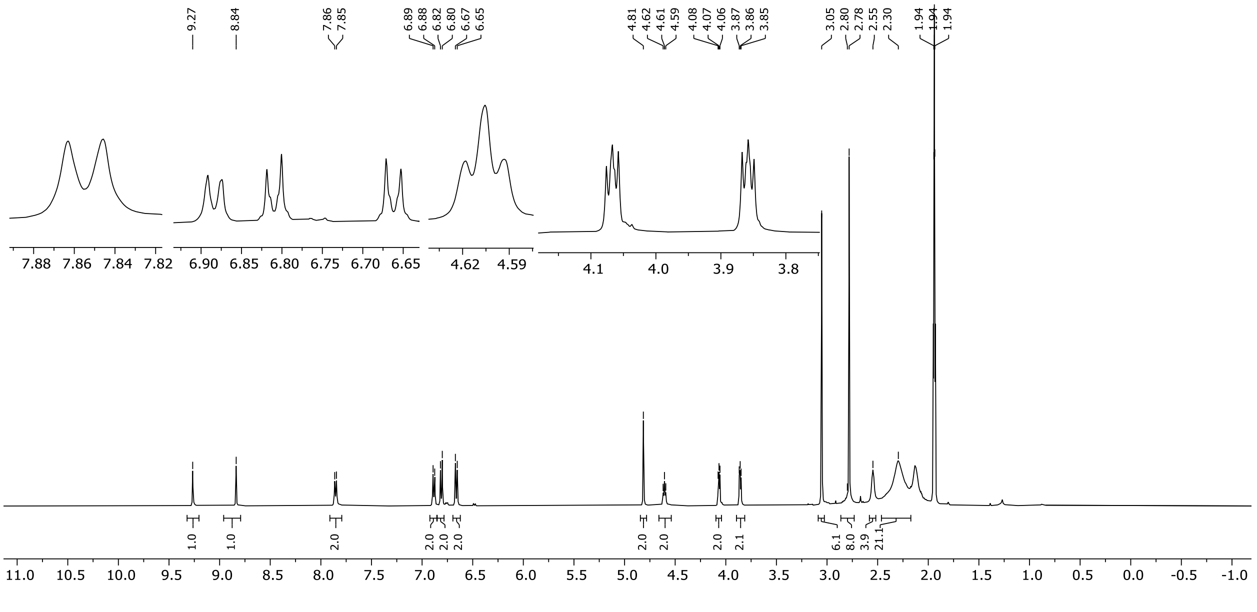

4-(6-(4-((2-(4-(Dimethylamino)phenoxy)ethoxy)methyl)-1*H*-1,2,3-triazol-1-yl)-9-vinyl-9*H*-purin-8-yl)-*N*,*N*-dimethylaniline (32)

^{1}H-NMR (500 MHz, $CDCl_3$) spectrum of compound 32:

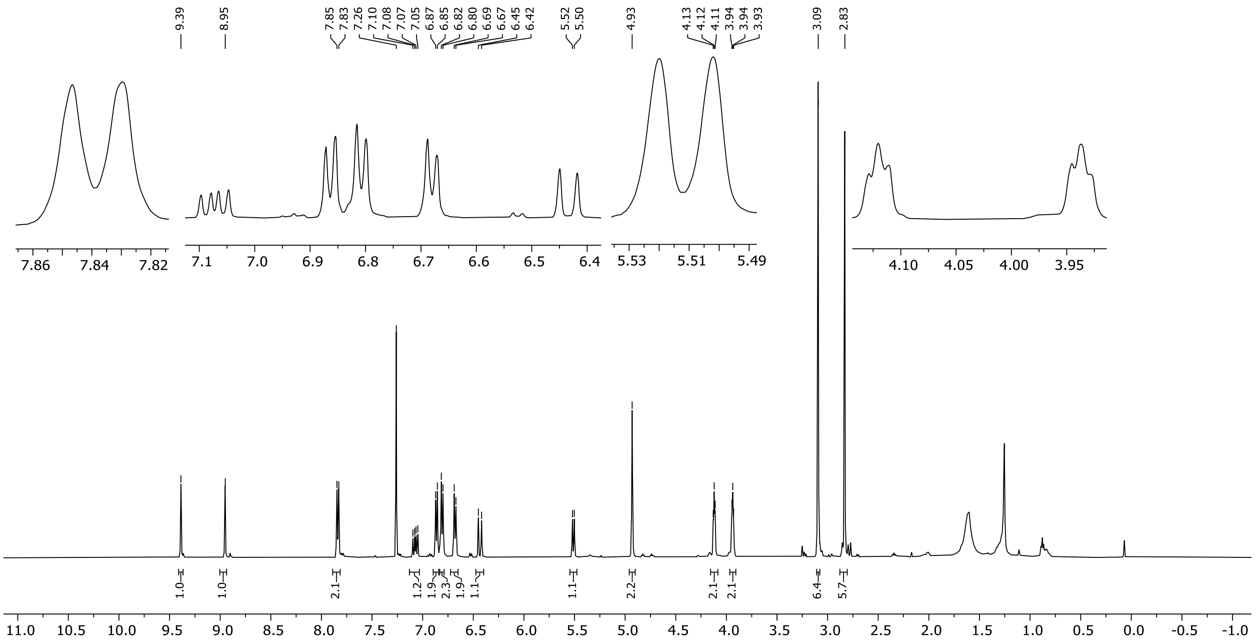

^{13}C-NMR (126 MHz, $CDCl_3$) spectrum of compound 32:

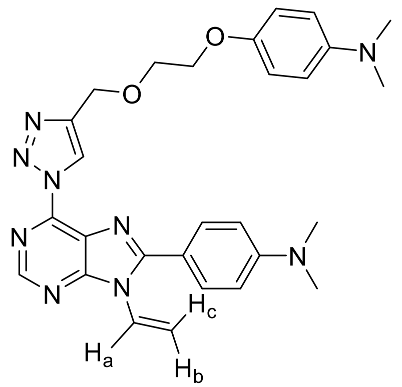

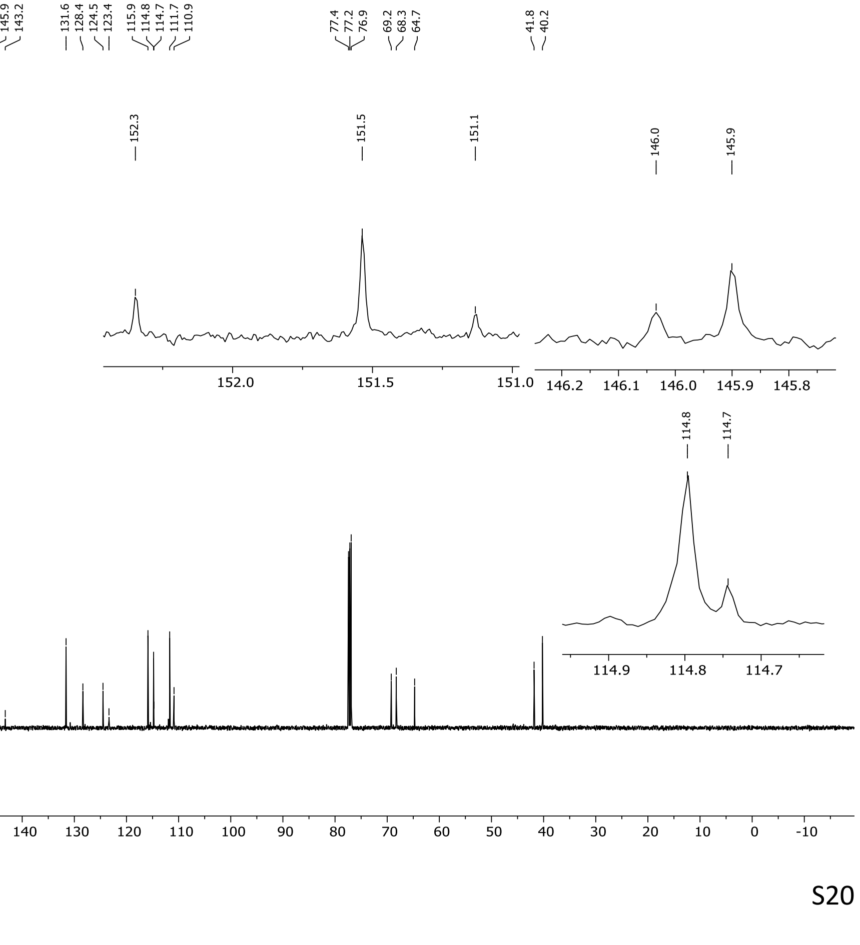

3. NMR titration experiment

An NMR titration example of compound **1** is described in this section.

In the NMR tube, compound **1** was dissolved in MeCN-d_3 (0.4 mL) and benzene (1 µL) was added to it as an internal standard. The amount of substance was calculated (Table S1).

Table S1. Amount of compound **1** in NMR tube.

**m_{Sample} =**	3.05	mg
**Purity =**	95.00	%
**M_{sample} =**	739.974	mg/mmol
**n_{Sample} =**	3.9157	µmol

A solution of $Cu(ClO_4)_2 \cdot 6\ H_2O$ in MeCN-d_3 with known concentration was prepared (Table S2).

Table S2. Calculation of concentration of a titrant.

**m_{Salt} =**	50	mg
**MW_{salt} =**	370.54	mg/mmol
**n_{Salt} =**	134.94	µmol
**$d_{Solvent}$ =**	844	mg/mL
**$m_{Solvent}$ =**	890	mg
**$V_{Solvent}$ =**	1.0545	mL
**$c_{Titrant}$ =**	0.12796	M

Then ^{1}H-NMR spectrum was taken (for 0 eq.). After that, the calculated amount of the titrant was added to the NMR tube (0.05 eq., 1.53 µL, Table S3), which was shaken vigorously before taking the subsequent ^{1}H-NMR spectrum. This procedure was repeated by adding the desired amount of titrant and taking the repeated spectra. All spectra were calibrated using a benzene signal at 7.37 ppm, then stacked, and their signal shifts were analyzed (Figure S1).

Table S3. Calculation of concentration of titrant

Entry	eq. of ion	$n_{Titrant\ needed}$, µmol	$V_{Titrant\ needed}$, µL	ΔV, µL
**1**	0.00	0.00	0.00	0.00
**2**	0.05	0.196	1.53	1.53
**3**	0.10	0.392	3.06	1.53
**4**	0.15	0.587	4.59	1.53
**5**	0.20	0.783	6.12	1.53
**6**	0.25	0.979	7.65	1.53
**7**	0.30	1.175	9.18	1.53
**8**	0.35	1.370	10.71	1.53
**9**	0.40	1.566	12.24	1.53
**10**	0.50	1.958	15.30	3.06
**11**	0.60	2.349	18.36	3.06
**12**	0.70	2.741	21.42	3.06
**13**	0.80	3.133	24.48	3.06
**14**	0.90	3.524	27.54	3.06
**15**	1.00	3.916	30.60	3.06
**16**	1.50	5.874	45.90	15.30

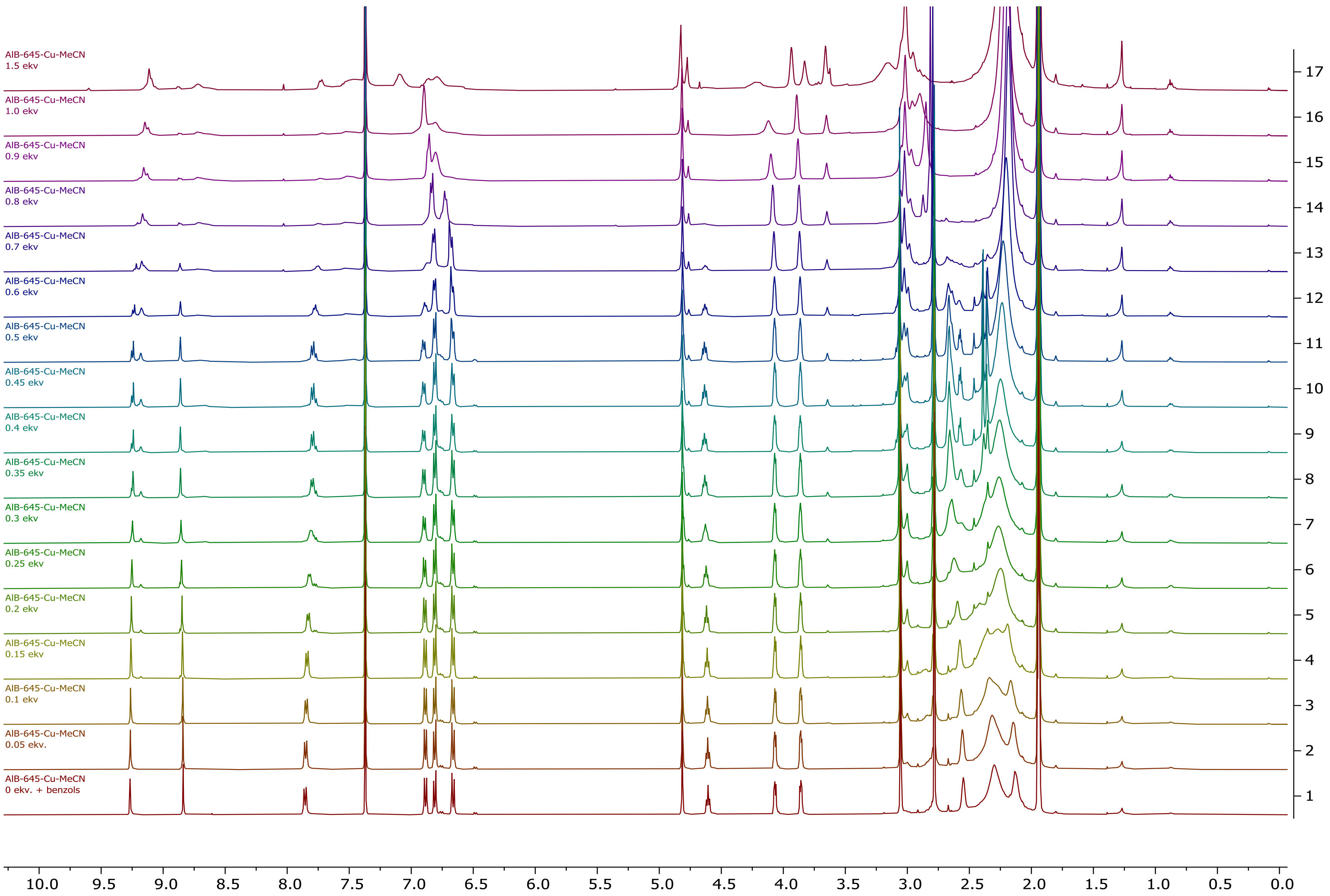

Figure S1. ^{1}H-NMR spectra (500 MHz) of titration experiments of compound **1** in MeCN-d_3, with $Cu(ClO_4)_2 \cdot 6\ H_2O$ as a Cu^{2+} source and benzene as an internal standard
